# Leucine-Based Pseudo-Proteins (LPPs) as Promising Biomaterials: A Study of Cell-Supporting Properties

**DOI:** 10.3390/polym15153328

**Published:** 2023-08-07

**Authors:** Mariam Ksovreli, Tinatin Kachlishvili, Tevdore Mtiulishvili, Giorgi Dzmanashvili, Tatuli Batsatsashvili, Knarita Zurabiani, David Tughushi, Temur Kantaria, Lili Nadaraia, Levan Rusishvili, Olivier Piot, Christine Terryn, Pavel Tchelidze, Ramaz Katsarava, Nina Kulikova

**Affiliations:** 1 Institute of Cellular and Molecular Biology, Agricultural University of Georgia, 0159 Tbilisi, Georgia; 2 Institute of Chemistry and Molecular Engineering, Agricultural University of Georgia, 0159 Tbilisi, Georgia; 3 Institute of Physical Material Science and Materials Technologies, Technical University of Georgia, 0159 Tbilisi, Georgia; 4 Carl Zeiss Scientific and Education Center, New Vision University, 0159 Tbilisi, Georgia; 5 Department of Morphology, Faculty of Exact and Natural Sciences, Ivane Javakhishvili Tbilisi State University, 0159 Tbilisi, Georgia; 6 BioSpecT Unit, University of Reims Champagne-Ardenne, 51100 Reims, France; 7 Faculty of Healthcare, East European University, 0159 Tbilisi, Georgia

**Keywords:** pseudo-proteins, polymeric scaffolds, biomimetics

## Abstract

Scaffold-based systems have become essential in biomedical research, providing the possibility of building in vitro models that can better mimic tissue/organic physiology. A relatively new family of biomimetics—pseudo-proteins (PPs)—can therefore be considered especially promising in this context. Three different artificial leucine-based LPP films were tested in vitro as potential scaffolding materials. In vitro experiments were performed using two types of cells: primary mouse skin fibroblasts and a murine monocyte/macrophages cell line, RAW264.7. Cell adhesion and cell spreading were evaluated according to morphological parameters via scanning electron microscopy (SEM), and they were assessed according to actin cytoskeleton distribution, which was studied via confocal laser microscopy. Cell proliferation was evaluated via an MTT assay. Cell migration was studied using time-lapse microscopy. SEM images for both types of cells demonstrated prominent adhesion and perfect cell spreading on all three LPPs. Analyses of actin cytoskeleton organization revealed a high number of focal adhesions and prominent motility-associated structures. A certain stimulation of cell proliferation was detected in the cases of all three LPPs, and two of them promoted macrophage migration. Overall, our data suggest that the LPPs used in the study can be considered potential cell-friendly scaffolding materials.

## 1. Introduction

Over the past several decades, thousands of in vitro tissue models have emerged on the surfaces of scaffolding polymer films or semiliquid cell carriers. These scaffold-based systems have become essential in biomedical research, offering the possibility of building in vitro models that can better mimic tissue/organic physiology and pathology [1,2,3]. Therefore, the synthesis and clinical use of novel biopolymers, as well as the development of relevant scaffold-based platforms, offer promising paths toward satisfying the growing demand for scaffold-based systems in regeneration and transplantation medicine [1,2,3].

Naturally occurring biodegradable polymers with high affinities for tissues like collagen, which is widely used in biomedical applications, have some important limitations. They demonstrate batch-to-batch variation and represent risks of disease transmission and immune rejection [4,5,6]. The latter limitation is connected to the molecular architecture of collagen (and proteins in general). It is known that in a protein molecule, α-L-amino acids (AAs) have a so-called “head-to-tail” orientation (assuming that the α-amino group is the “head” and the α-carboxyl groups are the “tail”). This natural proteinaceous architecture of macromolecules containing only amide NH-CO (peptide) groups is easily recognized by the body’s immune system [4,5,6].

Taking into account the above-mentioned limitations of natural polymers, the development of a broad family of artificial degradable polymers (DPs) provides new perspectives on the field of tissue engineering. These synthetic DPs have important advantages: they represent no risk of disease transmission and instigate a moderate immune response ranging from low to zero [4]. On the other hand, most artificial polymers have other limitations: a lack of “physiological” chemical bonds providing affinities for tissues (e.g., amide NH-CO links similar to peptide bonds) and a decreased nutritional potential compared to natural polymers as most artificial polymers do not produce AAs during degradation [4].

Therefore, artificial biomimetic polymers, which on the one hand, biodegrade into α-L-amino acids (AAs), and on the other hand, demonstrate degrees of immunogenicity ranging from low to zero, combine the advantageous features of both natural and artificial polymers. Clearly, if the chemical and physical features of an artificial biopolymer are close to those of organic biopolymers, this makes the biopolymer “cell-friendly” with respect to cell adhesion/spreading, cell movement, cell orientation, cell-to-cell communication, and organization into a perfectly sufficient cellular society. In this context, pseudo-proteins (PPs), a relatively new family of biomimetics, can be considered [4].

PPs have non-proteinaceous architectures, i.e., the orientations of the AAs in their polymeric backbones are not “head-to-tail” but “tail-to-tail” or “head-to-head”. Their structures are achieved by using two types of dimerized AAs as basic building blocks, either (A) diacyl-*bis*-α-amino acids or (B) *bis*-(α-amino acid)-alkylene diesters [4,7]. These artificial biomaterials have high potential for practical use in both fundamental and applied research.

**Figure 1:**
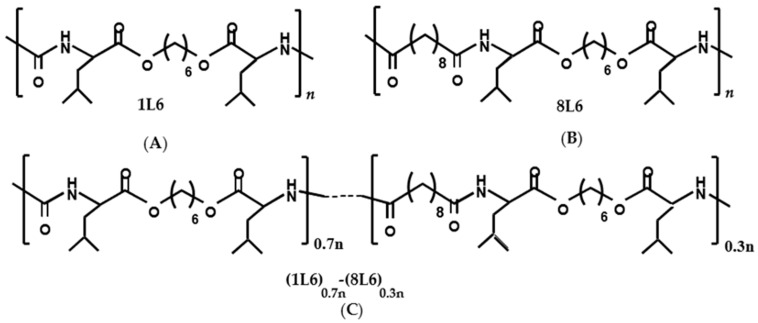
Chemical structures of the pseudo-polymers used in the study: (**A**) 8L6 (composed of L-leucine, 1,6-hexanediol, and sebacic acid); (**B**) 1L6 (composed of carbonic acid (1) and L-leucine (L); (**C**) copolymer of 1L6 and 8L6.

We previously showed that nanoparticles produced from two different artificial LPPs (Figure 1), 8L6 (composed of L-leucine, 1,6-hexanediol, and sebacic acid) and 1L6 (composed of carbonic acid (1) and L-leucine (L)), demonstrate low levels of cytotoxicity with different stable cell lines [8]. As the same LPPs can be used for the creation of thin, solid films, we presumed that due to their high degrees of biocompatibility, they can be considered potential scaffolding materials. Additionally, a copolymeric LPP made from 1L6 and 8L6 (70 mol% 1L6 and 30 mol% 8L6) has been used (Figure 1). It was important to evaluate the cell-supporting properties of the given LPPs. For this reason, the physiological and morphological parameters of cells growing on the polymeric substrates were evaluated. Moreover, taking into consideration that the topological properties of a substrate affect the physiology of the cells it supports [9,10,11], the surface topologies of the LPPs were studied at a sub-micro level. The results revealed that all three LPPs differ according to their levels of porosity. Therefore, certain dissimilarities in the effects of the LPPs on cell physiology that were observed during the research might reflect these differences in their topologies. It should be stressed that all three LPPs promoted a tight affinity and good spreading of the growing cells. Therefore, the use of the suggested LPPs as cell-supporting substrates can be a novel research strategy in a broad range of biomedical areas.

## 2. Materials and Methods

### 2.1. Reagents and Materials

Dulbecco’s Modified Eagle Medium (DMEM) and Fluoroshield histology mounting medium were purchased from Sigma Aldrich (St. Louis, MO, USA); fetal bovine serum (FBS) (Capricorn Scientific), paraformaldehyde (ROTH), glutaraldehyde, 25% solution (Sigma Aldrich, St. Louis, MO, USA), Phalloidin iFluor 647 (Abcam, Cambridge, UK); 4′,6-diamidino-2-phenylindole (DAPI), dimethyl sulfoxide (DMSO), phosphate-buffered saline (PBS), and 2.5% Trypsin-EDTA all were purchased from Santa Cruz Biotechnology (Dallas, TX, USA).

The murine macrophage cell line, RAW264.7 (ATTC TIB-71), was purchased from the American Type Cell Culture Collection (ATCC, Manassas, VA, USA). HeLa cells stably expressing histone H2B-GFP were kindly provided by Prof. P.Tchelidze, who had used them in his previous research [12,13,14,15,16]. Primary mouse skin fibroblasts (pMSFs) had been previously prepared at our laboratory according to the protocol described in [17], and the early passages of pMSFs were stored at −86 °C. These cells were thawed and passaged for the current experiments.

### 2.2. Synthesis of L-leucine-based PPs: 1L6, 8L6, and a Copolymer (1L6)_0.7_-(8L6)_0.3_

The key bis-nucleophilic monomer for synthesizing all three PPs is a di-*p*-toluenesulfonic acid salt of bis-(L-leucine)-1,6-hexylene diester, L6. It was prepared via the direct condensation of an amino acid (2.0 mol) with a diol (1.0 mol) in the presence of *p*-toluenesulfonic acid monohydrate (2.0 mol) in a refluxed hydrophobic organic solvent (in this case, cyclohaxane), according to the published procedure [18]. The L6 monomer precipitated in the organic solvent as a white powder after the reaction had completed and was filtered off, dried, and recrystallized from water; m.p. 190–192 °C, which coincides with the reported data.

To synthesize the poly(ester urea) class 1L6 PP, 6.89 g (10 mmol) of the L6 monomer was suspended in a beaker in 150 mL of water, and 4.24 g (40 mmol) of anhydrous sodium carbonate was added, stirred at room temperature for 30 min, and cooled to 10 °C. In parallel, 0.9892 g (10/3 mmol) of triphosgene (98% from Sigma-Aldrich), used as a bis-electrophilic monomer, was dissolved in 35 mL of dichloromethane (DCM) and cooled to 5 °C. The first solution was placed into a reactor for interfacial polycondensation, and the second solution was quickly added and stirred for 20 min. The DCM layer was then separated, dried over anhydrous Na_2_SO_4_, and filtered. The obtained solution was evaporated, and the resulting polymeric film was dried in a vacuum at 45 °C until a constant weight was achieved (yield: 94%).

The synthesis of the poly(ester amide) class 8L6 PP was carried out according to the procedure above, using 2.3914 g (10 mmole) of sebacoyl chloride (99% from Sigma-Aldrich) as a bis-electrophilic monomer instead of 0.9892 g of triphosgene. The 8L6 PP was separated as described above.

The synthesis of the poly(ester urea-*co*-ester amide) class (1L6)_0.7_-(8L6)_0.3_ copolymeric PP was carried out according to the same procedure, using a mixture of 0.6924 g (7/3 mmole) of triphosgene and 0.7174 (3 mmole) of sebacoyl chloride as a bis-electrophilic monomer. The PP (1L6)_0.7_-(8L6)_0.3_ was separated as described above.

All three PPs were purified via re-precipitation from a 10% ethanol solution in a 20-fold excess of distilled water. The precipitated rubbery polymers were filtered off and dried in a vacuum at 45 °C until constant weights were achieved.

The weight average molecular weight (M_w_) and dispersity (*Đ*) of each polymer were determined via a GPC machine (Waters Associates, Inc., Milford, CT, USA). Styragel columns in DMF, HR4, HR3, and HR0.5 (all 7.8 mm × 300 mm) were equipped with a high-pressure liquid chromatography pump (Waters 1525 Binary HPLC) and a Waters refractive index detector 2414 and UV detector (Waters 2487 dual absorbance detector, λ = 240 nm). A solution of LiBr (0.1 M) in DMF was used as the eluent. The injected volume was 100 µL, the sample concentration was 5.0 mg/mL, and the flow rate was 1.0 mL/min. The columns were calibrated using PMMA standards. PP 1L6: M_w_ = 146,600, *Đ* = 1.65; PP 8L6: M_w_ = 58,800, *Đ* = 1.76; PP (1L6)_0.7_-(8L6)_0.3_: M_w_ = 86,200, *Đ* = 1.88.

### 2.3. Preparation of Polymeric Films from LPPs

A 7% solution of each of the LPPs in absolute ethanol was prepared. Using 96-well plates, 40 µL of LPP solution was distributed per well. The wells were air-dried in a laminar flow hood. Using glass cover slips, each slip was covered with 100 µL of LPP solution and air-dried in a laminar flow hood.

### 2.4. Cell Cultures

All three types of cells (pMSF, RAW264.7, and HeLa) were cultured under standard conditions (37 °C and 5% CO_2_) in DMEM supplemented with 10% fetal calf serum (FCS), 2 mM of L-glutamine, 50 U/mL of penicillin, and 50 μg/mL of streptomycin. At 80% confluence, the cells were harvested and seeded for the experiment on 96-well plates or coverslips which were pre-coated with LPPs or without LPP films. The cells were seeded at the following concentrations: 25 × 10^4^ per mL of RAW264.7 cells, 20 × 10^4^ per mL of HeLa cells, and 15 × 10^4^ per mL of pMSFs.

### 2.5. Scanning Electron Microscopy

An SEM analysis was applied for the following purposes: (a) In order to study the surface properties of the “naked” LPP films, focusing on micro-topography and morphometric parameters, and (b) to assess the “cell-friendly” features of the LPPs’ surfaces, particularly with respect to the stretching/spreading abilities of the adhesive and less-adhesive cellular types.

SEM samples were prepared using the following approach. The appropriate variants of the sub-confluent cell cultures of interest (COIs), growing on glass surfaces or the LPP polymer films, were selected in order that the morphology, density, and distribution of the cells were controlled under PMC. The COIs submitted to the SEM analysis were initially pre-fixed for 10 min with 4% PF, diluted in PBS solution, and then post-fixed (for 30 min) in a 2.5% glutaraldehyde solution prepared with the same buffer. Due to the solubility of the polymers in alcohol and acetone, no dehydration procedure was performed before they were coated with gold. Instead, the COIs settled on uncoated and coated coverslips were washed (3 × 5 min) with PBS and briefly rinsed with deionized water. The coverslips were fixed on a sample holder and air-dried for 30 min in a special hermetic container. Afterwards, the residual cellular water was evaporated inside a vacuum camera at 3.2–3.5 Pa, using a JEOL JEC3000FC/Auto Fine Coater. The coating was continued for 180 s at 40 mA to yield a reliable gold layer that completely covered the cell surfaces. Both the preliminary examination of the samples at low magnification and the registration of the images at low, medium, and high magnifications were performed using a JEOL JSM6510LV SEM at an accelerating tension of 20 kV and a working distance (WD) of 18–25 mm, while the samples were tilted at 35°–90°. For the quantitative analysis of the polymers’ micro- and nanopores via SEM, images were treated using ImageJ software (the initial scale bar was set automatically by the JEOL SEM control software). The final versions of the illustrations were ameliorated and collated in Photoshop and CorelDraw, respectively.

### 2.6. MTT Assay

An MTT assay was used to measure cellular metabolic activity as an indicator of cell viability, proliferation, and cytotoxicity. A 3-(4,5-dimethylthiazol-2-yl)-2,5-diphenyltetrazoliumbromide (MTT) assay [19] based on the ability of the mitochondrial dehydrogenase enzymes in metabolically active cells to cleave the tetrazolium rings of the pale yellow MTT and form a dark blue formazan crystal was conducted. The number of metabolically active cells is directly proportional to the level of the formazan product created. The MTT assay was performed according to the standard protocol described in [6]. Briefly, the cells were seeded in a 96-well plate at densities of 5 × 10^4^ RAW264.7 cells and 3 × 10^4^ pMSFs per well (200 μL of cells suspended in DMEM per well). For the control, the cells were seeded without a PP substrate, and for the blank controls, the wells were coated with PPs, and 200 μL of DMEM was added. After 24 h or 8 h of incubation, 10 μL of MTT (0.25 mg/mL) was added to each well and incubated with the cells for 3 h. Afterwards, the wells were dried out, and 100 μL of DMSO was added per each well to solubilize the formazan crystals. After shaking the wells in a dark environment for 10 min, the supernatant was removed into a new 96-well plate, and the absorbance at a wavelength of 450 nm was measured using a microplate spectrophotometer (ELx800 Absorbance reader, Biotek, Agilent Technologies, Dallas, TX, USA). The transferring step was necessary since the substrates of the PPs on the bottoms of the wells interfered with the optical density measurement.

### 2.7. Evaluation of Apoptosis with DiOC_6_

For the detection of apoptosis, the green lipophilic dye 3, 3′-dihexyloxacarbocyanine iodide (DiOC_6_) was used. DiOC_6_ is a cell-permeable, lipophilic, green fluorescent dye that is selective for the mitochondria of live cells [20,21]. For the apoptosis assay, the cells were mechanically detached from the wells using a culture media, washed with PBS, and stained with 40 nM of DiOC_6_. The cells were incubated for 15 min at 37 °C and analyzed via flow cytometry, using an Accuri C6 (BD, Franklin Lakes, NJ, USA). The fluorescence was excited using an argon laser (excitation wavelength: 488 nm) and analyzed in FL1 (wavelength: 530 ± 30 nm).

### 2.8. Evaluation of Cell Migratory Properties

For the examination of cell migration, the cells were seeded at a density of 3.5 × 10^5^ on each cover slip (with and without the PP films), and after 8 h or 24 h of incubation, time-lapse imaging was performed using a Nikon TMS inverted-phase contrast microscope with an AmScope MU300 digital camera, capturing a series of 60 images over a duration of 30 min. To analyze the data, “ImageJ” was utilized to convert the captured images into specialized formats, while an open-source software, “Cell Tracker v1.1.1” ref. [22], facilitated the manual tracking of the migration parameters (the total way of run, the average distance from the origin, and the average speed) for each individual cell.

### 2.9. Fluorescent Staining of F-Actin

For visualizing the actin cytoskeleton, we used a bicyclic peptide, Phalloidin, which binds to actin filaments (F-actin) [23]. The cells were seeded on coverslips that were pre-coated with LPPs or were without LPPs in the following concentrations: 3.5 × 10^5^ of pMSFs per sample, and 5 × 10^5^ of RAW264.7 cells per sample. After 24 h of incubation, the cells were fixed with 4% paraformaldehyde for 20 min, washed twice with PBS, and permeabilized with 0.1% Triton X-100/PBS for 5 min. The samples were washed twice and stained with Phalloidin-iFluor 647 (1 μg/mL) for 60 min. Afterwards, they were washed twice again and stained with DAPI (0.05 μg/mL) for 5 min. Finally, the samples were washed once more with PBS and placed in a drop of mounting medium (Fluoroshield, Sigma Aldrich, St. Louis, MO, USA). The samples were observed under a confocal laser microscope (LSM900). Phalloidin-iFluor 647 was detected at Ex/Em = 650/665 nm, and DAPI was detected at Ex/Em = 405 nm. We carried out the corresponding steps at optical zooms of ×200 and ×630 (objective: ×20 and ×63; ocular: ×10) using the following objectives: (i) Plan-Apochromat/×20/with a 0.8 M27 numerical aperture, and (ii) Plan-Apochromat/×63/1.4 Oil DIC M27.

### 2.10. Statistical Analysis

Data are reported as means ± standard errors of the mean (SEMs) of at least three independent experiments performed in triplicate. Student’s *t*-test was used to perform a statistical analysis of the differences among the experimental groups, and *p*-values < 0.05 were considered statistically significant.

## 3. Results

### 3.1. Evaluation of Metabolic Activity via MTT Assay

**Figure 2:**
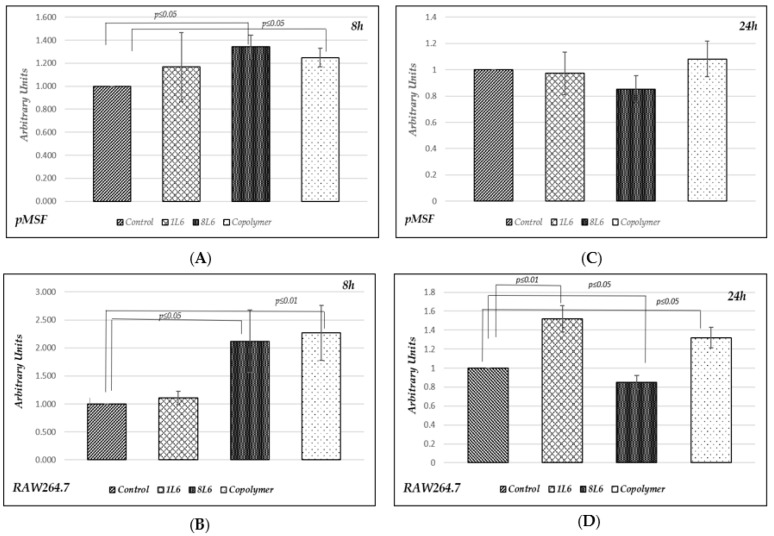
Metabolic activity according to MTT assay. Results are presented in arbitrary units compared to the control. (**A**) In pMSFs after 8 h of incubation (five independent experiments); (**B**) in RAW264.7 after 8 h of incubation (four independent experiments); (**C**) in pMSFs after 24 h of incubation (ten independent experiments); (**D**) in RAW264.7 after 24 h of incubation (eight independent experiments).

The 3-(4,5-dimethylthiazol-2-yl)-2,5-diphenyltetrazoliumbromide (MTT) assay [19] is based on the ability of a mitochondrial dehydrogenase enzyme in a metabolically active cell to cleave the tetrazolium rings of the pale yellow MTT and form a dark blue formazan crystal. The number of metabolically active cells is directly proportional to the level of the formazan product created. The MTT assay was used to measure cellular metabolic activity as an indicator of cell viability, proliferation, and cytotoxicity. Measurements were performed for both cell types (pMSFs and RAW264.7) at two time-points: after 8 h and after 24 h of cell growth (Figure 2). According to the obtained results, after 8 h of incubation, a statistically significant increase in metabolic activity (compared to the control) was detected for both cell types grown on 8L6 or the copolymer. There was no increase in metabolic activity in the case of 1L6; however, it should be stressed that there was no decrease, either (Figure 2A,B).

These data show that none of the three LPPs revealed any cytotoxicity, while increases in the levels of metabolic activity of the cells grown on 8L6 and the copolymer might indicate an increase in proliferation.

At a later time-point, after 24 h of incubation, the situation changed. For the fibroblasts (pMSFs), there were no statistically significant changes in metabolic activity compared to the control (Figure 2C), while for the macrophages (RAW264.7), the picture was different as increases in metabolic activity were registered for the cells grown on 1L6 and on the copolymer, while a certain decrease in metabolic activity was detected for the cells grown on 8L6 (Figure 2D). A decrease in metabolic activity can happen due to cytotoxicity; however, the reason could be different. As an increase in metabolic activity precedes proliferation, it might indicate that the peak in metabolic activity for these particular samples occurs at 8 h of growth and they actually proliferate at 24 h, but no activation of metabolic activity is detectable at this point. We presumed that cytotoxicity was not the cause in this case as no morphologically apoptotic cells were visible in these cell cultures. To check this, we decided to measure the level of apoptosis, and the outcome is presented in the next subsection of the results.

### 3.2. Measurement of Apoptosis Level Using DiOC_6_

The level of apoptosis was measured after 24 h of incubation for both types of cells, the pMSFs and the RAW264.7 line. A lipophilic dye, DiOC_6_, was used for the evaluation of apoptosis [20,21], and the results were registered via flow cytometry (Accuri, C6, BD).

**Figure 3:**
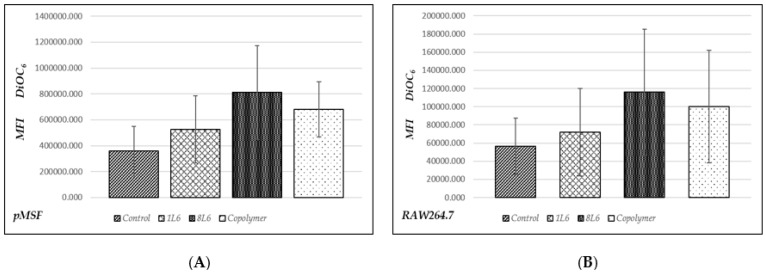
Evaluation of apoptosis level according to DiOC_6_ staining. On the vertical axis, the mean fluorescence intensity (MFI) of DiOC_6_ is shown. Staining was performed after 24 h of cell growth on the PP films (or without PPs in the control). (**A**) MFI of DiOC_6_ measured in fibroblasts (pMSF); (**B**) MFI of DiOC_6_ measured in macrophages (RAW264.7). Data from five independent experiments were summarized.

The data are presented in Figure 3. In the case of apoptosis, the mean fluorescence intensity (MFI) of the DiOC_6_ on the FL1 channel should decrease; however, it is clearly visible in Figure 3 there was no decrease in the MFI of DiOC_6_ for either cell type, indicating that there was no increase in the level of apoptosis for any of the three LPPs.

### 3.3. Morphological Evaluation of Apoptosis Using an H2B-GFP-Transfected HeLa Cell Line

The histone H2B-GFP-transfected HeLa cell line was used in our experiments to once again determine whether there was any induction of apoptosis, especially in the cells grown on 8L6 for which a decrease in metabolic activity was detected after 24 h of incubation. Due to nuclear fluorescence, this cell culture has been widely used in studies aimed at the detection of the early stages of apoptosis, which are recognized by changes in the nuclear shape and chromatin structure [24,25]. It would have also been easy to recognize an early response of the nucleus in the case of a possible polymer-induced cytotoxic and/or apoptogenic influence.

**Figure 4:**
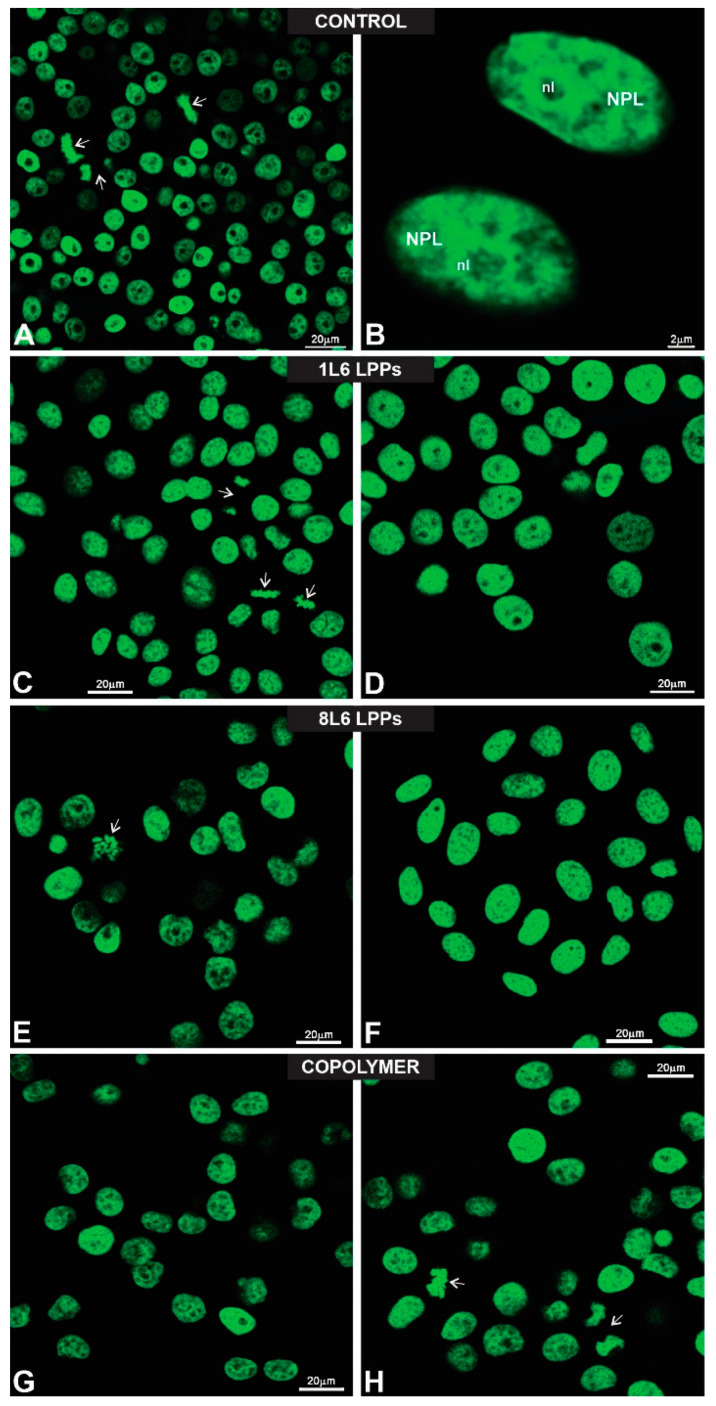
Gallery of fluorescent images showing HeLa human cell line stably expressing histone H2B-GFP tag within nuclei. These cells were settled on glass (control, **A**,**B**), 1L6 (**C**,**D**), 8L6 (**E**,**F**), and copolymer (**G**,**H**) surfaces. Note that all nuclei emitted bright green fluorescence. Moreover, note the number of mitotic cells (arrows; **A**–**C**). To present chromatin texture, nuclei were imaged under high magnification (**B**). Arrows are pointing to mitotic cells. NPL—nucleoplasm; nl—nucleolus.

In our experiments, histone H2B-GFP-transfected HeLa cells were grown on all three types of LPPs for 24 h, and their morphologies were then evaluated using laser confocal microscopy (Figure 4).

The fluorescent images show (Figure 4) that all polymers used in our study provided clearly relevant substrates for the histone H2B-GFP-transfected HeLa cells, and neither changes in the intensity of nuclear fluorescence nor alterations in the nuclear shape and chromatin structure that could be attributed to a cytotoxic effect were observed via fluorescent microscopy (Figure 4), thus confirming once again that all three LPPs reveal no toxicity.

### 3.4. Mitotic Index (MI) Calculation for RAW264.7 Cells

**Figure 5:**
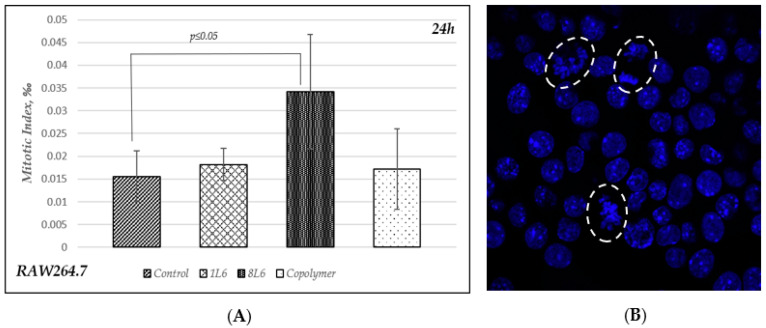
(**A**) Mitotic index calculated in control and in cells grown on LPPs after 24 h of incubation. Data from three independent experiments were summarized; 1000 cells were counted for each experiment. (**B**) A representative picture of RAW264.7 cells stained with DAPI. Mitotic nuclei are marked with ovals.

To carry out an additional evaluation of the physiological state of the RAW264.7 cells grown on LPP films for 24 h, we performed DAPI staining, which gave us an opportunity to reveal the morphology of the nuclei and count the number of mitotic cells. The MI was calculated for 1000 cells per sample (three independent experiments were performed), and the data are provided in Figure 5. As can be seen from the data, it appeared that the MI of the cells grown on the LPP 8L6 was significantly higher than the MI of both the control and the samples grown on the other two LPPs (Figure 5). This corresponds well to the suggested explanation for the MTT results (showing a decrease in metabolic activity for the cells grown on 8L6 for 24 h) that on this particular LPP film, the RAW264.7 cells became stimulated around 8 h of growth and that the increase in metabolic activity precedes proliferation, which becomes visible later, at the 24 h time-point, and at this time, no increase in metabolic activity can be seen.

### 3.5. A Study of the Migratory Properties of RAW264.7 Cells

**Figure 6:**
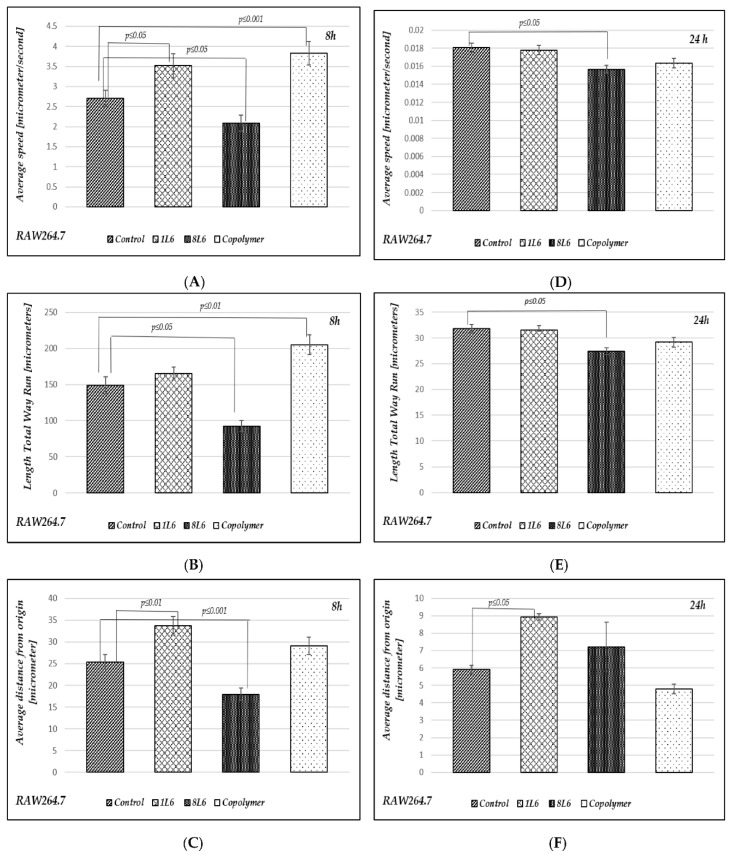
Evaluation of the RAW264.7 cells’ motility. (**A**) The average speed, calculated after 8 h of incubation; (**B**) the length of the total way of run, calculated after 8 h of incubation; (**C**) the average distance from the origin, calculated after 8 h of incubation; (**D**) the average speed, calculated after 24 h of incubation; (**E**) the length of the total way of run, calculated after 24 h of incubation; (**F**) the average distance from the origin, calculated after 24 h of incubation. Data from three independent experiments were summarized.

To investigate how the LPP substrates affect the cells’ migratory properties, the motility of the RAW264.7 cells was studied using time-lapse microscopy. Again, experiments were performed for two time-points, after 8 h and 24 h of incubation (Figure 6). For each sample, a series of 60 images were taken over a duration of 30 min. The captured images were analyzed via “Cell Tracker v1.1.1“ software [22], which evaluated the cells’ motility according to three different parameters: (1) The length of the total way of run; (2) the average distance from the origin; and (3) the average speed for each individual cell.

As can be seen from the results shown (Figure 6), the cells’ motility was more intense at the earlier 8 h time-point, which is natural as there was more space for the cells to move around at this point compared to the later 24 h time-point. According to all three parameters in the case of the 8 h time-point and according to two parameters in the case of the 24 h time-point, the cells’ motility was limited in the case of LPP 8L6 compared to the control (Figure 6). Taking into consideration that for this particular LPP, the MI was increased, we can speculate that more intensive proliferation in the population of cells grown on this particular substrate led to a certain limitation of the cells’ motility. For the other two LPPs, 1L6 and the copolymer, an opposite effect was observed: an increase in migratory ability was detected at the 8 h time-point. At the 24 h time-point, LPP 1L6 demonstrated an increase of cell migration according to only one parameter, the average distance from the origin, while for the copolymer, after 24 h of incubation, no stimulatory effect was registered compared to the control (Figure 6). Overall, we can summarize that to a certain extent, the LPPs affect cell mobility and this effect is LPP-specific, leading to a limited mobility for the cells grown on 8L6 and the opposite—increased mobility—for the cells grown on 1L6 and the copolymer.

### 3.6. Scanning Electron Microscopy (SEM)

#### 3.6.1. A Study of the LPP Films’ Surface Topologies

**Figure 7:**
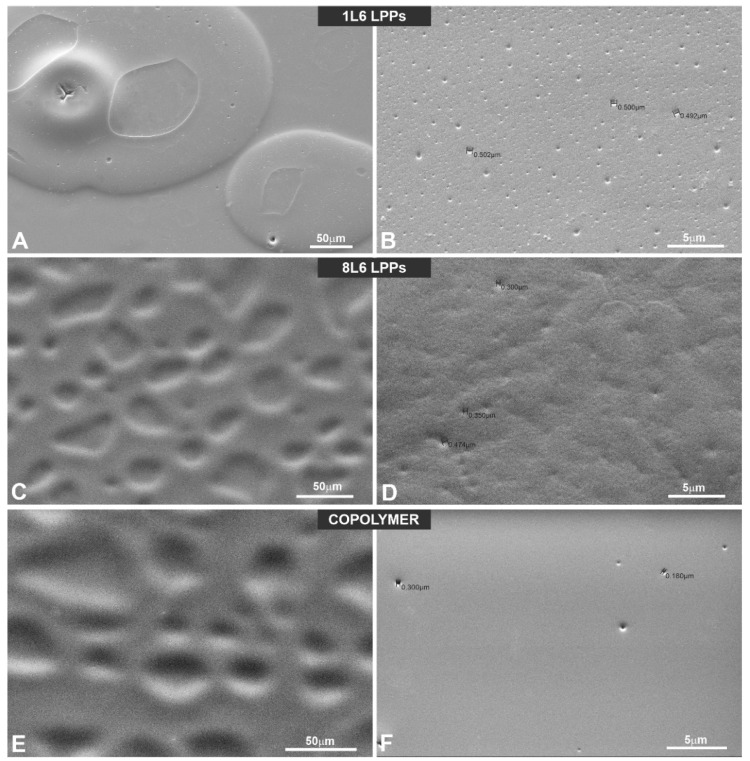
LPP surface characterizations via SEM. Representative SEM images of the LPPs’ surfaces: (**A**,**C**,**E**) ×400; (**B**,**D**,**F**) ×4000; (**A**,**B**) 1L6; (**C**,**D**) 8L6; (**E**,**F**) copolymer.

SEM was used to study the surface topology of each LPP at a sub-micro level. Others have shown [3,7,9,10,11,26,27] that a substrate’s surface topology affects the physiology of the cells it supports and that the most important factors are the topological parameters at the sub-micro level compared to the macro-level parameters [9]. Representative SEM images are presented in Figure 7.

The analysis of the surface topological parameters revealed that all three types of LPPs used in the experiments differ by porosity, which also corresponds to a general surface roughness to a certain extent (Table 1).

#### 3.6.2. A Morphological Assessment of pMSFs, RAW264.7 Cells, and HeLa Cells

Scanning electron microscopy (SEM) was used to evaluate the cells’ adhesion to the substrates based on their morphology. SEM images of the fibroblasts are presented in Figure 8, Figure 9, Figure 10 and Figure 11 in the main text and in Figure A1, Figure A2, Figure A3 and Figure A4 in Appendix A.

**Figure 8:**
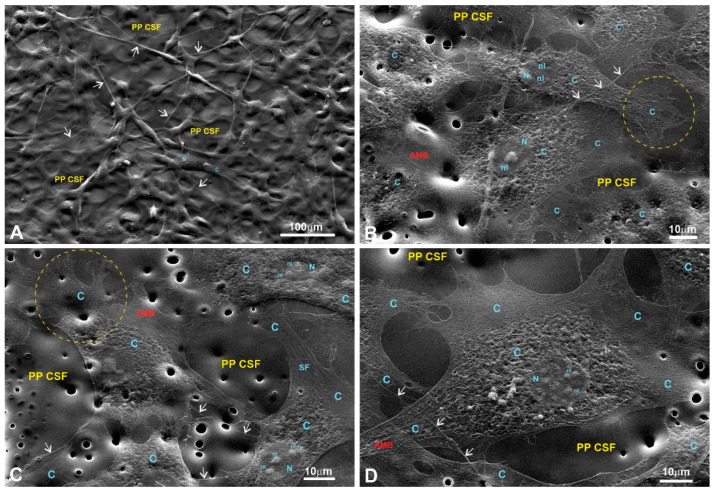
SEM images of fibroblasts growing on the surface of a PP 1L6 cell-supporting film (PP SCF). (**A**) Low magnification; (**B**–**D**) medium magnification. (**A**) A layer of properly stretched and chaotically growing cells with multiple cell-to-cell links established via extended primary and tiny secondary processes (arrows). (**D**) A strongly stretched cytoplasm is nicely visible. Motile cells form typical lamellipodia (outlined with dotted circles in (**B**,**C**)), long primary processes, and numerous secondary processes (arrows). All these features generate an impression of the cells’ tight attachment to the 1L6 LPP film. PP CSF—pseudo-protein cell-supporting film; C—cytoplasm; N—nucleus; nl—nucleolus; m—mitochondria; AMB—actin-myosin bundles.

**Figure 9:**
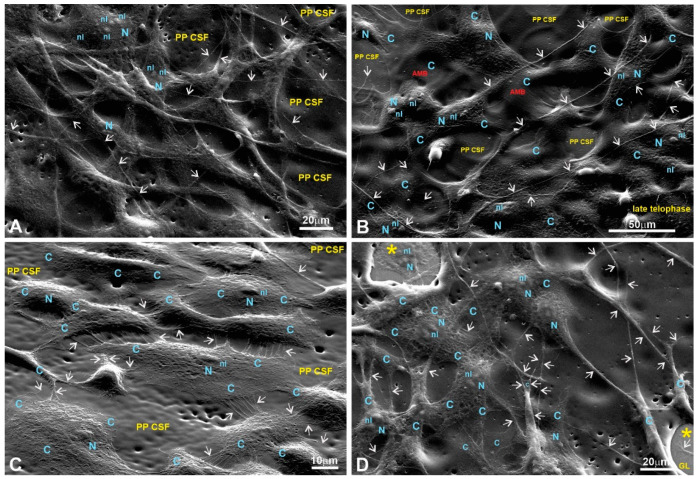
Fibroblasts growing on the surface of an 8L6 supporting film. (**A**,**B**) Low magnification; (**C**,**D**) medium magnification. (**A**,**B**) SEM images that demonstrate the network of properly stretched and chaotically growing cells. The cells exhibit major cell-specific features, including extended primary and secondary processes (arrows). Polymer-free areas, “holes” in LPP’s coating, where cells are settled on a glass surface are marked with star. Note, cells “climbing” from the glass onto the polymer, being tightly attached similarly to the glass surface and to 8L6 LPP’s surface. PP CSF—pseudo-protein cell-supporting film; C—cytoplasm; N—nucleus; nl—nucleolus; m—mitochondria.

**Figure 10:**
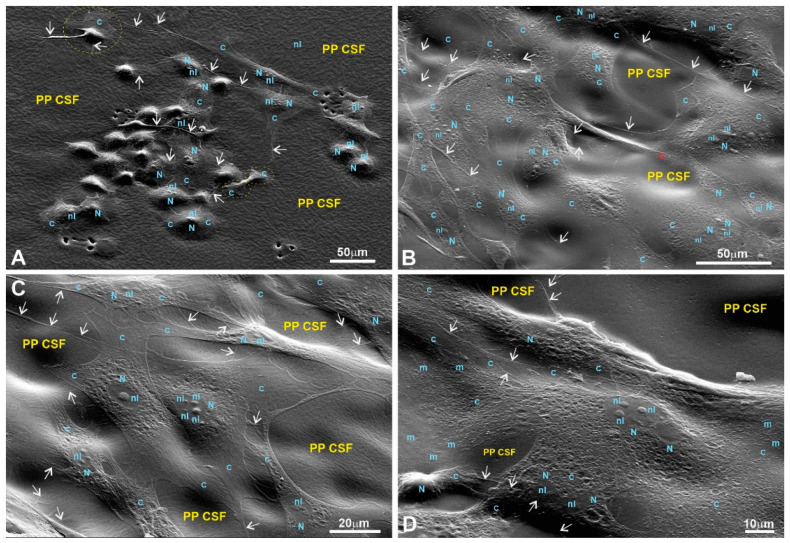
pMSFs growing on the surface of a copolymer supporting film. (**A**,**B**) Low magnification; (**C**,**D**) medium magnification. (**A**,**B**) Low magnification images demonstrate that similar to the 1L6 and 8L6 PP films, the copolymer facilitates the proper stretching and tight adherence (**B**) of the pMSFs. Meanwhile, even at the lowest magnification (**A**), motile cells are readily recognizable as they produced typical leading edges/fronts. In addition, the arrows on (**A**–**D**) indicate multiple primary and secondary processes, most probably representing the morphological counterpart of the tight adherence to substrate. PP CSF—pseudo-protein cell-supporting film; C—cytoplasm; N—nucleus; nl—nucleolus; m—mitochondria.

**Figure A1:**
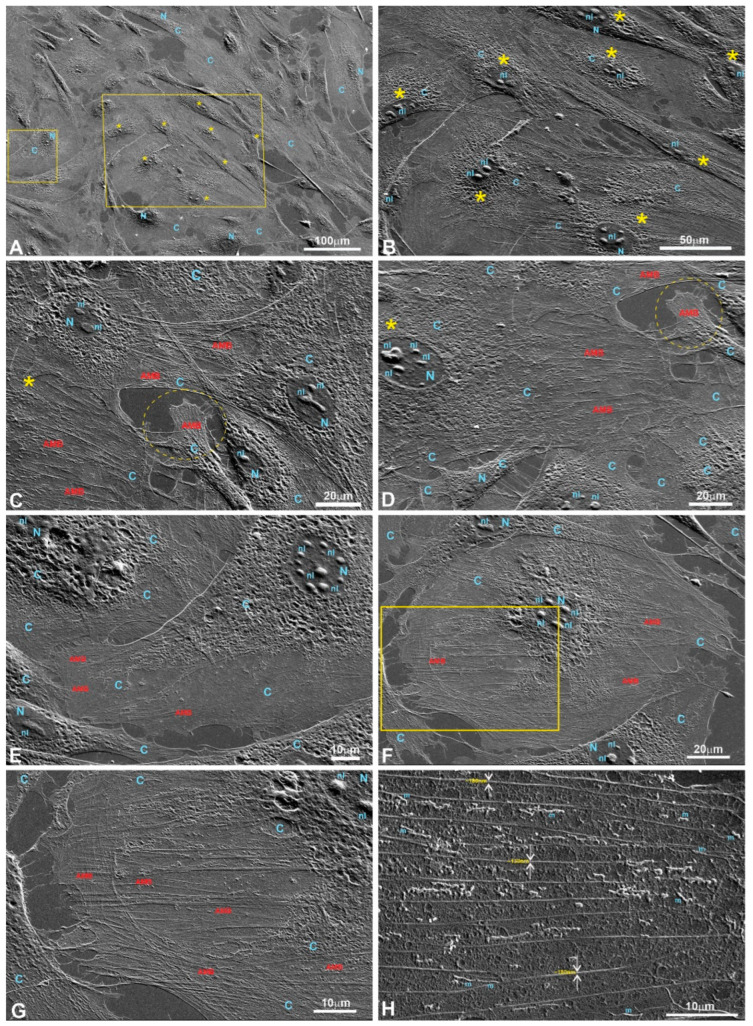
SEM images of fibroblasts growing on a glass surface. (**A**,**B**) Low magnification; (**C**–**F**) medium magnification; (**G**,**H**) high magnification. (**A**) At low magnification, it became clear that chaotically grown cells that form more or less dense layers are tightly associated via numerous cell-to-cell contacts; (**B**) the region shown in the large rectangle on A. Note that corresponding cells (marked with stars) reveal a properly stretched cytoplasm (**C**) with a profoundly structured interior and a well-defined nucleus (**N**) with numerous bulged nucleoli (nl), and structurally strictly different perinuclear and peripheral zones of the cytoplasm are readily recognizable even at a low level of magnification. Long processes that connect individual cells and their groups are also present. (**C**–**F**) Even at medium magnifications, it becomes clear that the distinctly visible stretched fibers that run inside the “empty” peripheral cytoplasm (**D**), which correspond to the region shown in the small rectangle in **A**), can be organized in parallel rows or chaotically oriented in different directions which often cross each other. (**F**) In addition, medium magnification has demonstrated that properly stretched fibroblasts reveal typical end-feet (outlined with dotted circles in **C**,**D**), leading edges, (**E**) and profound tripartite structures of the cellular interiors (**D**–**F**). Moreover, note the ovoid nuclei regularly containing more than one nucleoli of drastically different sizes and shapes. In some cells, the number of nucleoli can reach up to 10 (**D**); (**G**) Part of the cytoplasm shown in the rectangle in (**F**), revealing peripheral cytoplasm with bundles of stretched fibers crossing each other at a level of high magnification. Multiple processes that establish cell-to-cell communication are distinctly seen. (**H**) High-magnification image demonstrating a nearly parallel order of 0.1–0.2 µm cords (arrows) that correspond to stretching fibers and “worm”-like mitochondria (m). C—cytoplasm; N—nucleus; nl—nucleolus; m—mitochondria; AMB—actin-myosin bundles.

The SEM images of fibroblasts taken at low and medium magnifications for both controls and the experimental samples grown on LPPs reveal properly spread and chaotically distributed pMSFs that form loose networks throughout the substrates (Figure A1A,B (control); Figure 8A (1L6); Figure 9A,B (8L6); Figure 10A (copolymer)).

SEM images taken at a higher magnification reveal the presence of cytoskeletal fibers that run in different directions and cross over each other or even create cords that are 0.1–0.2 µm thick and arranged in parallel rows (Figure A1H (control); Figure 8C,D (1L6); Figure 9C,D (8L6); Figure 10C,D (copolymer)).

**Figure 11:**
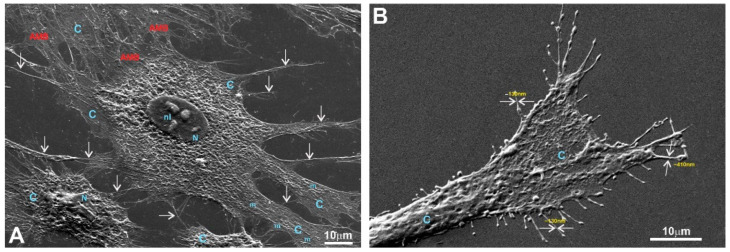
SEM image of a fibroblast growing on a glass surface. The fibroblast shows a “hairy” appearance due to numerous radially emanating primary processes. (**A**) Medium magnification; (**B**) high magnification. On (**A**), a tight network made of intermittent primary and secondary processes (arrows) is clearly visible; (**B**) the terminal part of the primary process with flat end-feet. Note that multiple secondary processes of 0.1–0.5 µm diameters (arrows) have tiny local expansions producing a specific “node”-like appearance. C—cytoplasm; N—nucleus; nl—nucleolus; m—mitochondria; AMB—actin-myosin bundles.

It should be noted that multiple motility-related structures—lamellipodia and filopodia—were visible on the SEM images (in the control and in all three LPPs) (Figure A1C,D (control); Figure 8B,C (1L6); Figure 9C,D (8L6); Figure 10C,D (copolymer)). Both types of protrusions establish multiple contacts with the substrate and neighboring cells. The filopodia protrusions were characterized as extremely long and more or less flattened processes (Figure 11A) and tiny secondary processes with diameters ranging between 0.1 and 1 µm (Figure 11B). Frequently, the tips of these processes form flat and roundish edges that are tightly attached to the substrate (Figure 11B). Due to numerous radially organized primary and secondary processes, some fibroblasts acquire a peculiar “hairy” appearance (Figure 11A).

**Figure 12:**
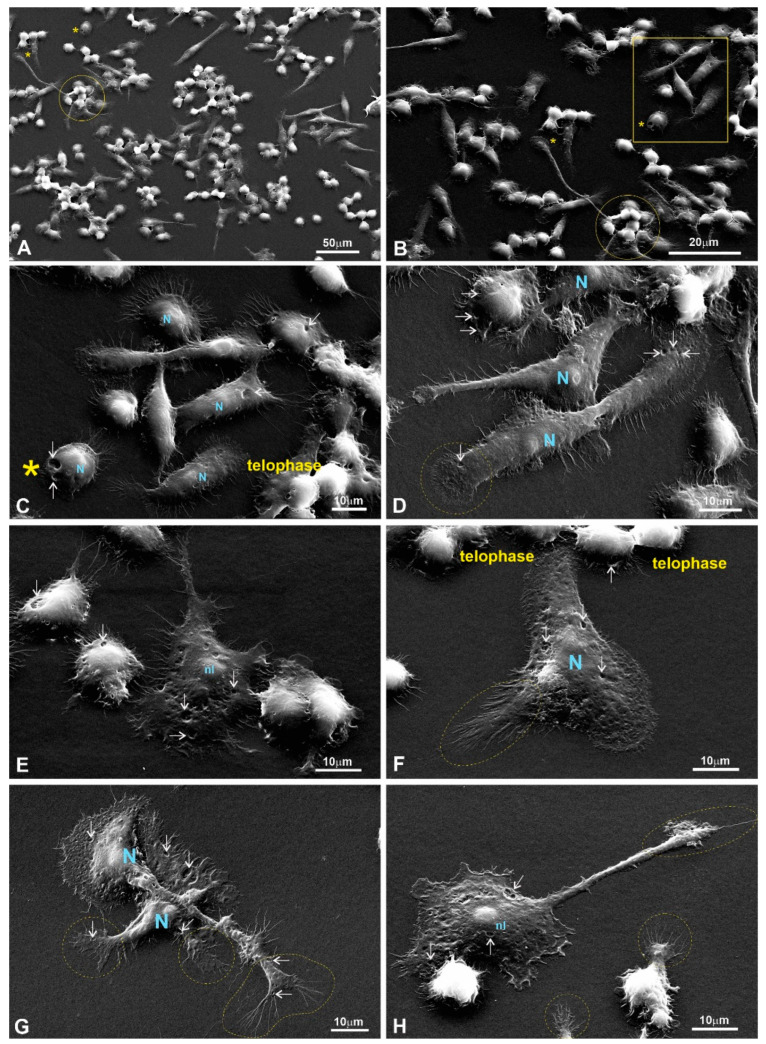
RAW264.7 macrophages growing on a 1L6 PP surface. (**A**,**B**) Low magnification; (**C**–**F**) medium magnification; (**G**,**H**) high magnification. Different subpopulations: bulged, flattened, and motile spindle-like elongated and motile macrophages were chaotically intermittent or gathered in groups of closely associated cells (outlined with solid circles in (**A**,**B**)). More or less flattened and motile cells produce numerous primary processes that can interconnect individual cells with grouped ones (marked with a star on (**A**) and a star outside of a rectangle in (**B**)). The macrophages in (**C**) correspond to the group outlined with the rectangle in (**B**). Corresponding bulged cells that reveal two depressions on their surfaces (arrows) are marked with stars (located inside the rectangle in (**B**)). All cells imaged at medium magnification abundantly produced secondary processes, and thus acquired a “hairy” cell appearance (**C**–**F**). Lamellipodia and end-feet that intensively produce secondary protrusions are outlined with dotted circles (**D**) and an ellipse (**F**). Bulged and flattened cells form numerous surface depressions (marked with arrows). (**G**,**H**) Two flattened, motile cells that actively produced typical leading edges with prominent end-feet primary processes (outlined with dotted circles and an ellipse), all abundantly spreading out with secondary protrusions. Moreover, visible on (**G**) are bulged cells that produced numerous secondary processes, growing from more flattened peripheral cytoplasm (outlined with a dotted circle). PP CSF—pseudo-protein cell-supporting film; C—cytoplasm; N—nucleus; nl—nucleolus.

To summarize what we observed for the pMSFs grown on LPPs, at low magnification, no differences were detected in the morphology or network-like organization of the cells between fibroblasts growing on the surfaces of the conventional substrates and all versions of the LPP films. The cells growing on the LPP scaffolds revealed branched or elongated and extremely sprawled appearances that were similar to the control. The corresponding SEM images demonstrate typically sprawled cells with stretching grades and ultrastructures indistinguishable from the conventional substrates. When focusing on cell periphery and especially the cell–polymer interface, it becomes clear that secondary processes, which are emitted from the primary processes, form multiple and very tight links with all types of LPP films used and establish intimate contact with neighboring cells (Figure 10C–E; Figure 11A,B; Figure 12C–H). Similarly, as in the control, in the fibroblasts cultured on the surfaces of LPP films, the ultrastructural organization of the cytoplasm is clearly visible at high magnification.

**Figure 13:**
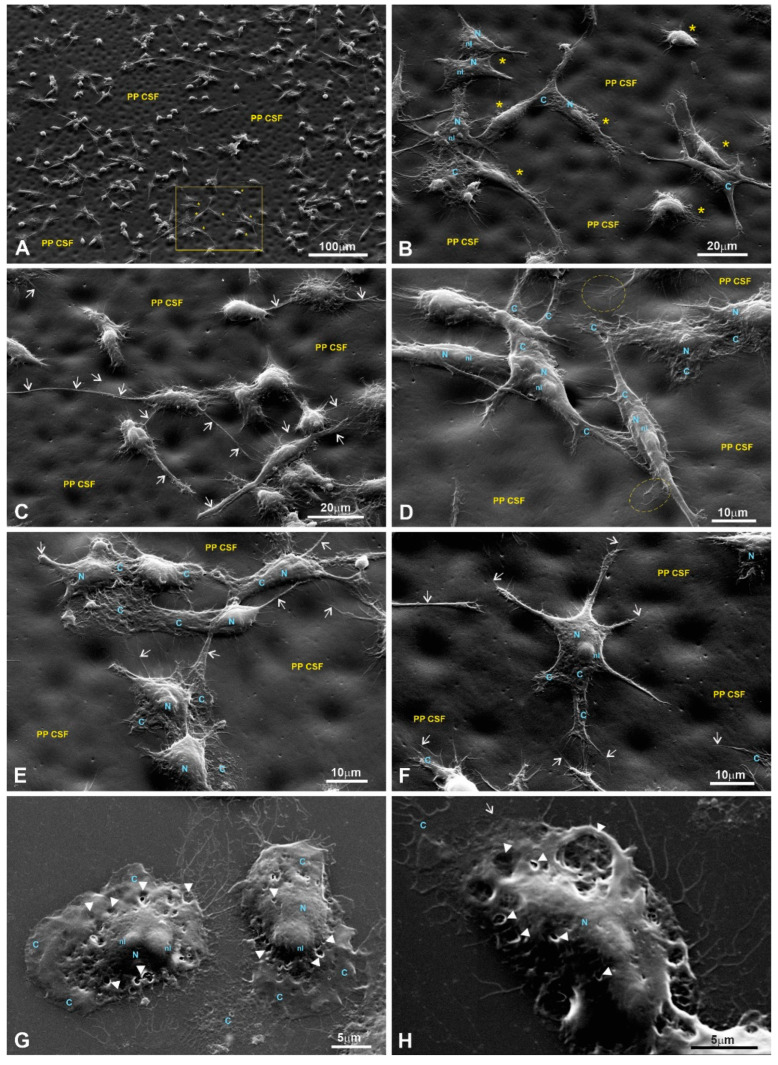
RAW264.7 macrophages growing on the 8L6 LPP film. (**A**) Low magnification; (**B**–**F**) medium magnification; (**G**,**H**) high magnification. Group of macrophages in (**B**) correspond to those shown in the rectangle in (**A**). Identical cells are marked with stars. Arrows in (**C**–**F**) indicate long primary processes, while end-feet in (**D**) are outlined with dotted circles. In turn, lamellipodia, leading edges, primary processes, as well as end-feet produce numerous secondary protrusions. Note the star-like cells located separately (**F**) that also produce numerous processes, running from radially emanating primary processes or directly from the peripheral cytoplasm. At a high level of magnification (**G**,**H**), numerous intensively branching secondary processes growing from extremely stretched lamellipodia become especially well pronounced. It is clearly visible that the surfaces of macrophages form numerous depressions of drastically different diameters (marked with arrowheads) that correspond to active endocytosis or phagocytosis. PP CSF—pseudo-protein cell-supporting film; C—cytoplasm; N—nucleus; nl—nucleolus.

**Figure 14:**
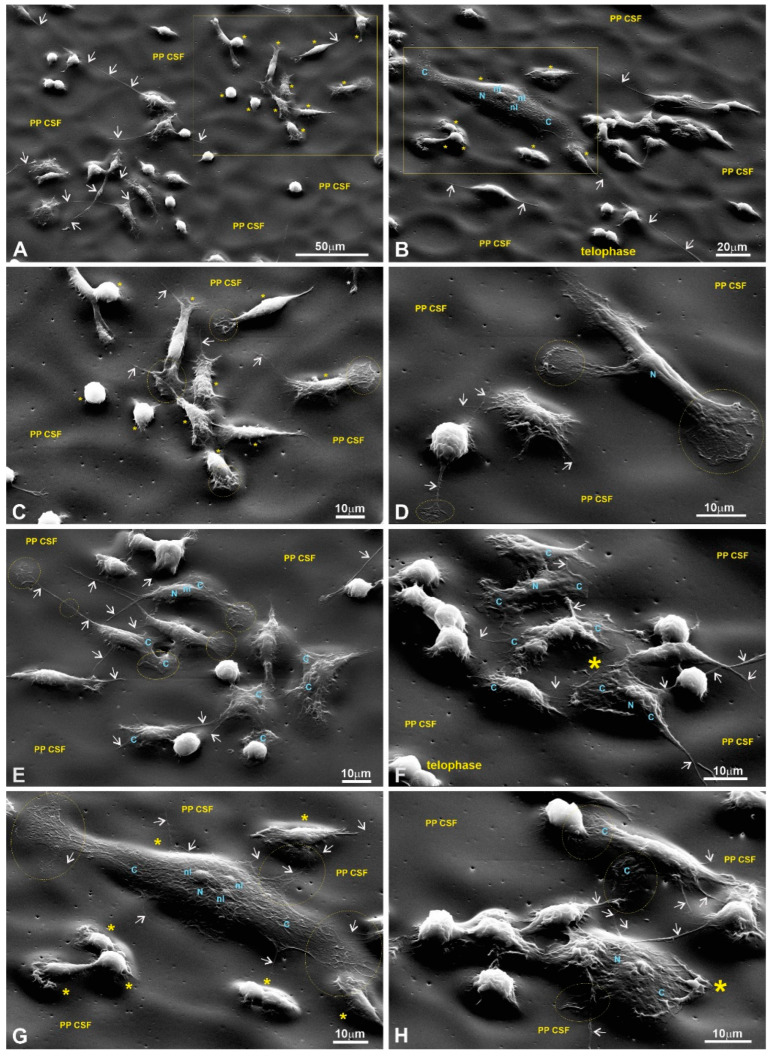
RAW264.7 macrophages growing on a copolymer surface; images taken at low (**A**,**B**), medium (**C**–**F**), and high (**G**,**H**) magnifications. The group of macrophages in (**C**) corresponds to cells shown in the rectangle in (**A**), while macrophages within the rectangle in (**B**) were imaged at a high magnification in (**G**). In all images, arrows indicate long primary processes that intensively produce secondary processes, while lamellipodia, leading edges, and end-feet are outlined with dotted circles (**C**–**E**,**G**,**H**). The macrophage marked with a star in (**F**) reveals a clearly motile cell morphology. Meanwhile, a flattened, large macrophage with typical end-feet formed by both poles (**G**), as well as the macrophage marked with a star as a well-stretched cell (**H**), exhibit pronounced nuclear and bulged nucleolar territories. PP CSF—pseudo-protein cell-supporting film; C—cytoplasm; N—nucleus; nl—nucleolus.

**Figure A5:**
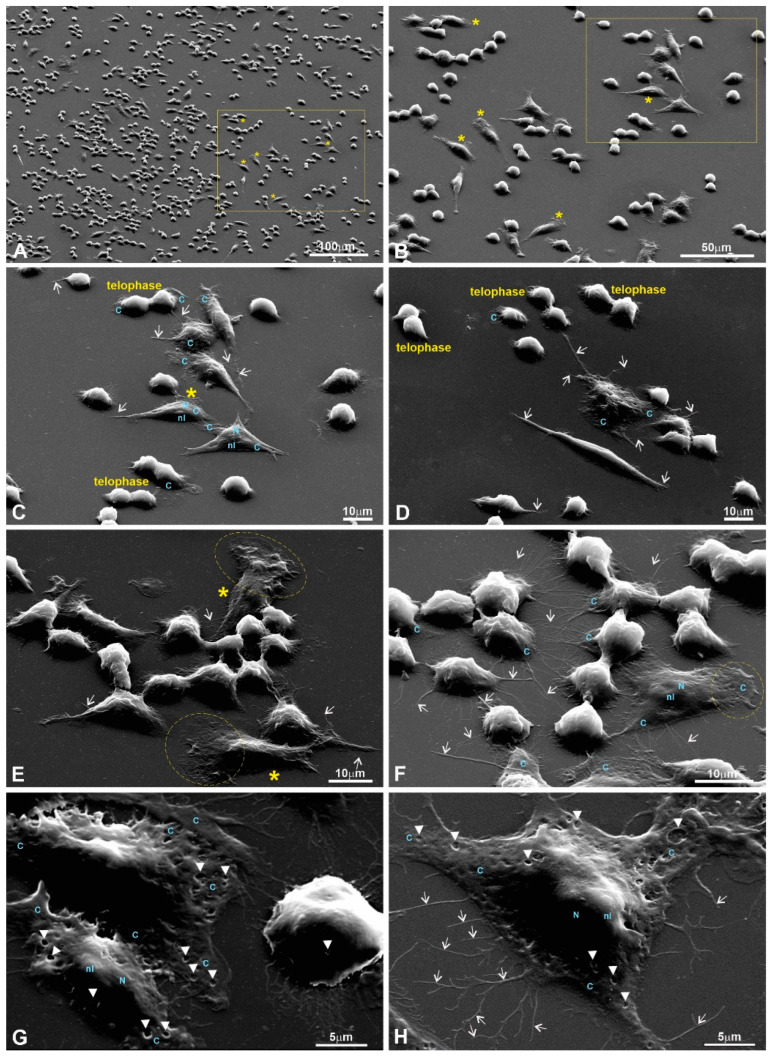
RAW264.7 cells growing on a glass surface, taken at low (**A**,**B**), medium (**C**–**F**), and high (**G**,**H**) magnifications. Among the chaotically growing macrophages (**A**), two morphologically distinct subpopulations are easily identified (**B**): (i) The major group consists of roundish/oval macrophages with characteristically bulged shapes. These cells appear less stretched in order that the nucleus and nucleolus could not be recognized. (ii) The minor group comprises profoundly flattened, spindle-like, elongated, and motile cells that reveal prominent lamellipodia (**B**). The macrophages in (**B**) correspond to the group of cells outlined with a rectangle in (**A**), taken at a higher magnification (the corresponding cells are marked with stars); the cells in (**C**), imaged at medium magnification, correspond to the rectangle in (**B**). Arrows indicate primary and secondary processes. Numerous arrowheads in (**G**,**H**) indicate cell surface depressions that could be presumed to be morphological reflections of active endocytosis/phagocytosis. C—cytoplasm; N—nucleus; nl—nucleolus.

The morphological features of the macrophages (RAW264.7 cells) cultured on the surfaces of the conventional substrates and/or on the surfaces of the LPPs used are displayed in Figure A5 (control) and Figure 12, Figure 13 and Figure 14 (LPPs). A precise visualization of the ultrastructural interplay between the RAW264.7 cells and different substrates was obtained via SEM imaging (Figure 12, Figure 13 and Figure 14). Detailed views of RAW264.7 cells growing on the LPP surfaces reveal that the level of adhesion for each of the three LPPs was the same as the level of adhesion for the control (see a detailed description provided in Figure A5 and Figure 12, Figure 13 and Figure 14 legends).

An additional type of cell cultured on the LPPs was the H2B-GFP-transfected HeLa cell line, which we initially chose for the experiments due to the cells’ nuclear fluorescence, which gave us an opportunity to easily monitor nuclear and chromatin morphologies.

**Figure A6:**
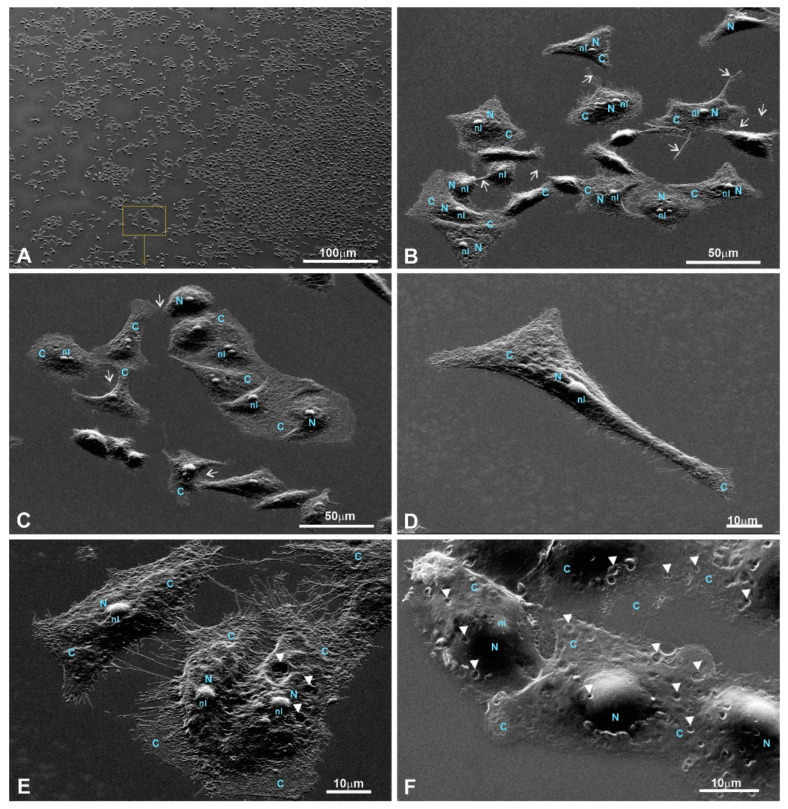
Images of H2B-GFP-transfected HeLa cells growing on a glass surface, taken at low (**A**,**B**), medium (**C**,**B**), and high (**E**,**F**) magnifications. (**A**) Chaotically growing HeLa culture at low magnification. Meanwhile, the group of cells shown in the rectangle corresponds to those presented in (**C**) at medium magnification. (**B**) HeLa cells shown at slightly higher magnification. Properly stretched and bulged cell subpopulations are visible. The arrows in (**B**) indicate long primary processes with numerous secondary ones. (**C**,**D**) At medium magnification, strongly stretched cells reveal well-delineated nuclear compartments with prominent nucleoli. The arrows indicate secondary protrusions. (**D**) A separately located HeLa cell with abundant secondary processes protruding from the primary ones. (**E**,**F**) Due to numerous secondary processes, properly stretched cells acquired a “hairy” appearance. At high magnification in strongly stretched HeLa cells, the nuclear zones with prominent nucleoli become especially profound. C—cytoplasm; N—nucleus; nl—nucleolus.

**Figure A7:**
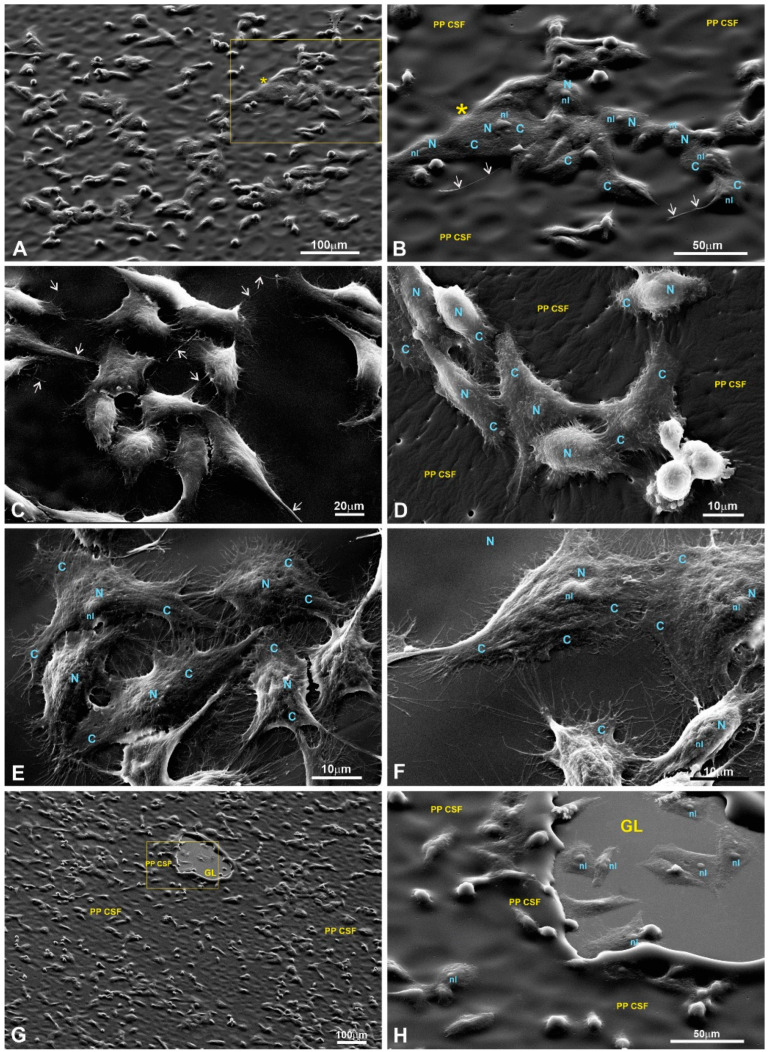
H2B-GFP-transfected HeLa cells growing on the 1L6 LPP. (**A**,**B**,**G**,**H**) Images taken at low (**C**–**E**), medium, and (**F**) high levels of magnification. The group of HeLa cells in (**B**) corresponds to the cells shown in the rectangle in (**A**) (identical cells are indicated with stars). The arrows in images (**B**,**C**) mark long primary processes that intensively produce secondary processes. Meanwhile, a flattened, large macrophage with typical end-feet formed by both poles (**G**), as well as a well-stretched cell marked with a star (**H**), both exhibit pronounced nuclear and bulged nucleolar territories. (**E**,**F**) The apparent “hairy” appearance of HeLa cells establishes multiple cell-to-cell contacts with each other. (**G**,**H**) These images present the region of the “heterogeneous” polymer, shown in the rectangle in (**G**). The same region was then imaged at a slightly higher magnification (**H**). Here, we can simultaneously observe HeLa cells positioned on polymer and glass surfaces, as well as cells located on the border between these two types of substrates. Importantly, regardless of the substrate’s nature and the position of the cells, their larger parts were properly stretched. As in previous cases, these images generate an impression of “climbing” cells. PP CSF—pseudo-protein cell-supporting film; C—cytoplasm; N—nucleus; nl—nucleolus.

**Figure A8:**
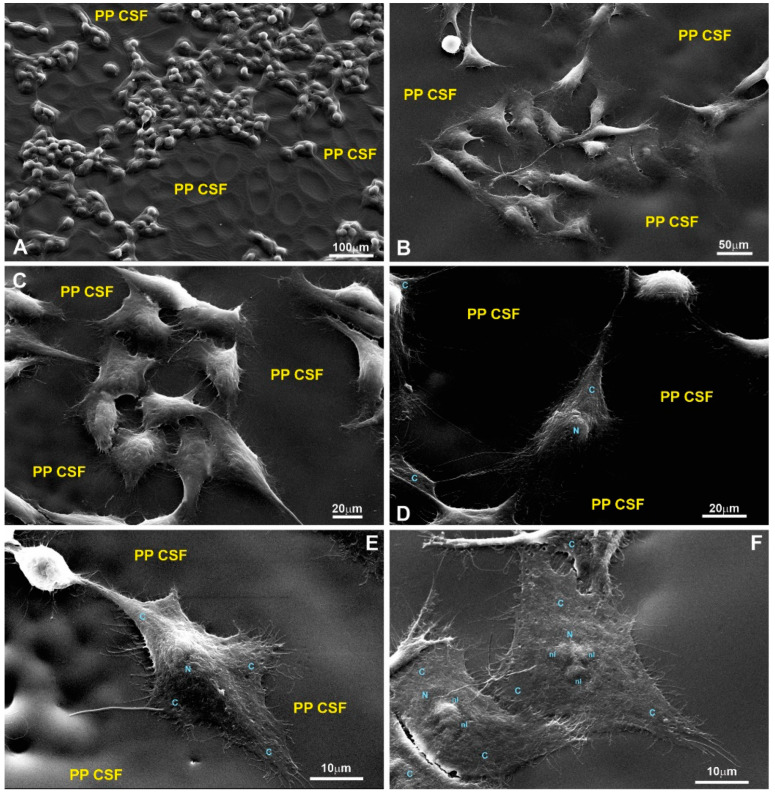
H2B-GFP-transfected HeLa cells growing on the 8L6 LPP. (**A**,**B**) Images taken at low magnification; (**C**,**D**) images taken at medium magnification; (**E**,**F**) images taken at high magnification. (**B**–**F**) Major cell-specific features (described in all previous cases), such as long primary processes and secondary protrusions, were easily recognizable. Due to the abundance of secondary processes that established multiple cell-to-cell links, the HeLa cells acquired a “hairy” appearance. Note the number of properly stretched HeLa cells in which the nuclear zones with bulged nucleoli are clearly seen (**B**,**F**). PP CSF—pseudo-protein cell-supporting film; C—cytoplasm; N—nucleus; nl—nucleolus.

**Figure A9:**
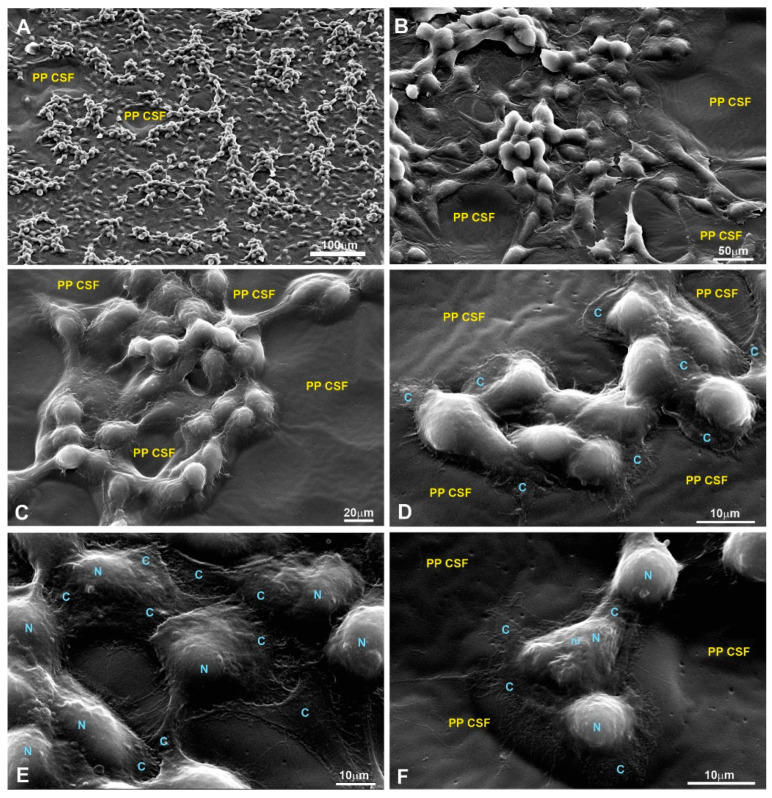
H2B-GFP-transfected HeLa cells growing on the copolymer LPP. (**A**,**B**) Images taken at low magnification; (**C**,**D**) images taken at medium magnification; (**E**,**F**) images taken at high magnification. When imaged at a low magnification, the HeLa culture exhibited not only extremely chaotic growth but also distribution in the form of three distinct cellular subpopulations (**A**). These subpopulations looked like: (i) Cellular groups that comprised more flattened cells; (ii) groups of properly stretched cells; (iii) groups of clearly bulged cells with a tightly linked appearance (**B**). It should be stressed that even the bulged-shape cells maintained the ability to produce intensively secondary processes, growing from a flattened peripheral cytoplasm (**D**). (**E**,**F**) The confirmed impression that all subpopulations were tightly anchored to the substrates, most probably using secondary processes to achieve strong anchorage. PP CSF—pseudo-protein cell-supporting film; C—cytoplasm; N—nucleus; nl—nucleolus.

For this particular cell line, SEM images were also obtained, showing a firm adhesion of the GFP-transfected cells to all types of LPP substrates (Figure A6, Figure A7, Figure A8 and Figure A9). Accordingly, the SEM control defined that the GFP-transfected cells were similarly and properly anchored to the glass surface (Figure A6B–F) and to all types of PP films (Figure A7B–H; Figure A8B–H; Figure A9D–F. A closer look at the cell boundaries (including those of a leading edge) reveals well-spread primary processes that are tightly linked to the substrate and their extensions (Figure A7E,F; Figure A8C–F; Figure A9D–F).

Using a detailed view of the cellular boundaries of the cells growing on all the LPP variants, we registered that the peripheral cytoplasm was quite properly spread and linked to the substrate via multiple primary and secondary processes (Figure 12 and Figure 13). At the same time, actively moving macrophages are readily recognizable as they regularly reveal typical lamellipodia (Figure 12D–H; Figure 13D–H; Figure 14B-H). As a rule, numerous primary processes created a “hairy” cell appearance, as revealed at medium and high magnifications (for example, see Figure 12C–F and Figure 13G,H). In turn, secondary processes produced by both primary processes and lamellipodia were also strongly spread and created an impression of their fusion with the LPPs (Figure 12C–H; Figure 13C–H; Figure 14C–H). Apparently, similar to the macrophages growing on the glass, the cells settled on the LPPs retained their active endocytosis/phagocytosis abilities as they also reveal numerous focal depressions of the cell surface (Figure 13G,H). Altogether, as the morphological analysis of the RAW264.7 cells’ growth on the LPPs confirm, these films provide substrates that are as “friendly” for macrophages as the conventional substrates.

### 3.7. Actin Cytoskeleton Distribution in pMSFs and RAW264.7 Cells

**Figure 15:**
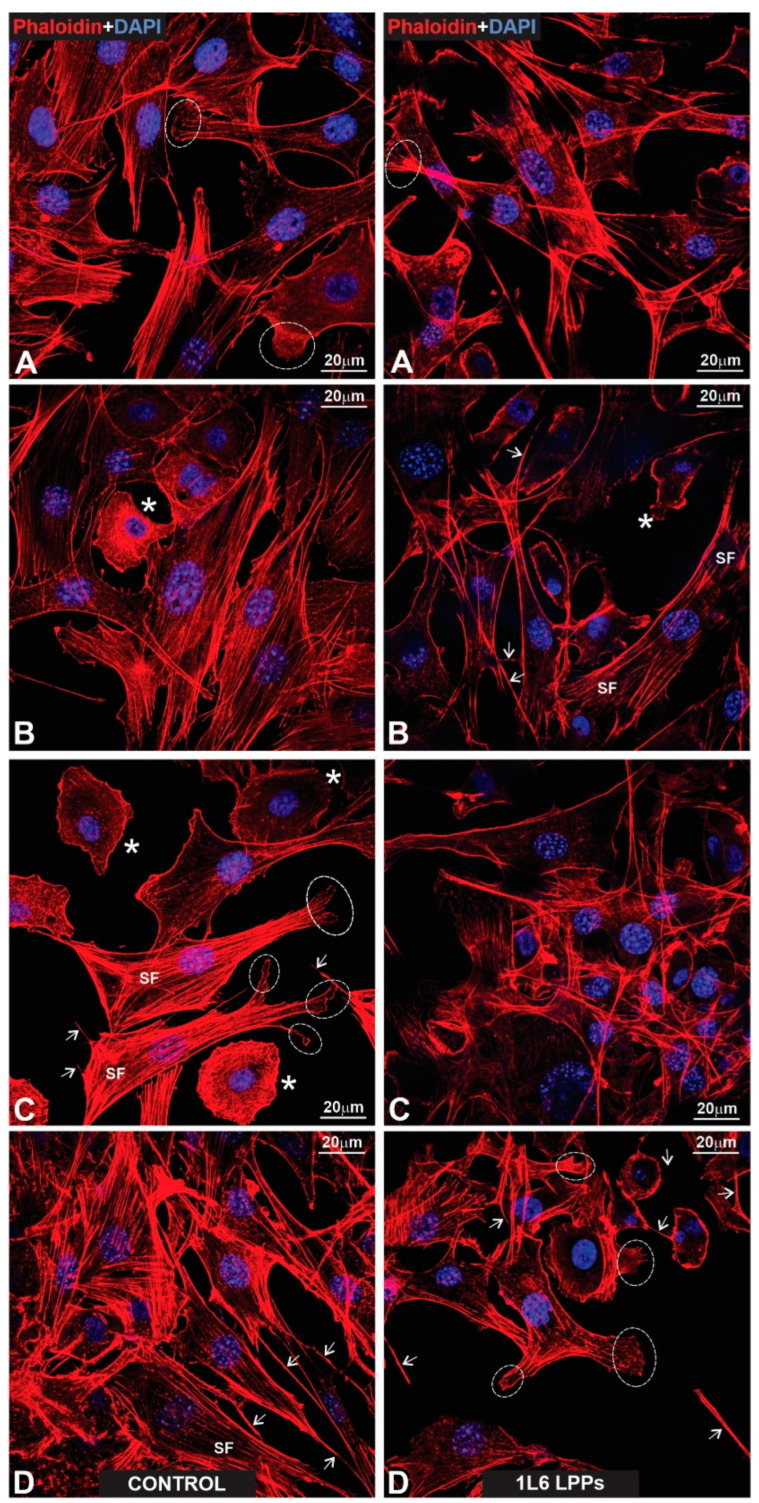
(**Left**: (**A**–**D**)) Gallery of fluorescent control images presenting fibroblasts growing on a glass surface that were specifically labeled for actin microfilaments using phalloidin (red). The nuclear label (blue) was obtained via DAPI counterstaining. All cells reveal a properly stretched appearance, exhibiting strongly developed stress fibers (SF; **A**–**D**). Note the prominent organization order of the stretched fibers in which their bundles form nearly parallel rows. Stars (**B**,**C**) indicate motile and properly stretched cells, both producing large lamellipodia. End-feet are outlined with dotted circles (**A**,**C**). The arrows in (**C**,**D**) indicate long primary processes with growing secondary processes. (**Right**: (**A**–**D**)) Fluorescent images presenting pMSFs that grow on the surface of 1L6 LPP. The same labeling as in the case of fibroblasts growing on a glass surface is used. All cells were properly stretched (**A**–**D**) and contained profound stress fibers (SF). The star in (**B**) marks a properly stretched cell that exhibits large lamellipodia. The end-feet are outlined with dotted circles (**A**,**C**). Long primary processes that produce secondary sprouts are marked with arrows (**B**). Note that in (**C**,**D**), very long and abundant primary processes emanating from different cells acquire complex network-like appearances by crossing each other and running on the surfaces of cells.

**Figure 16:**
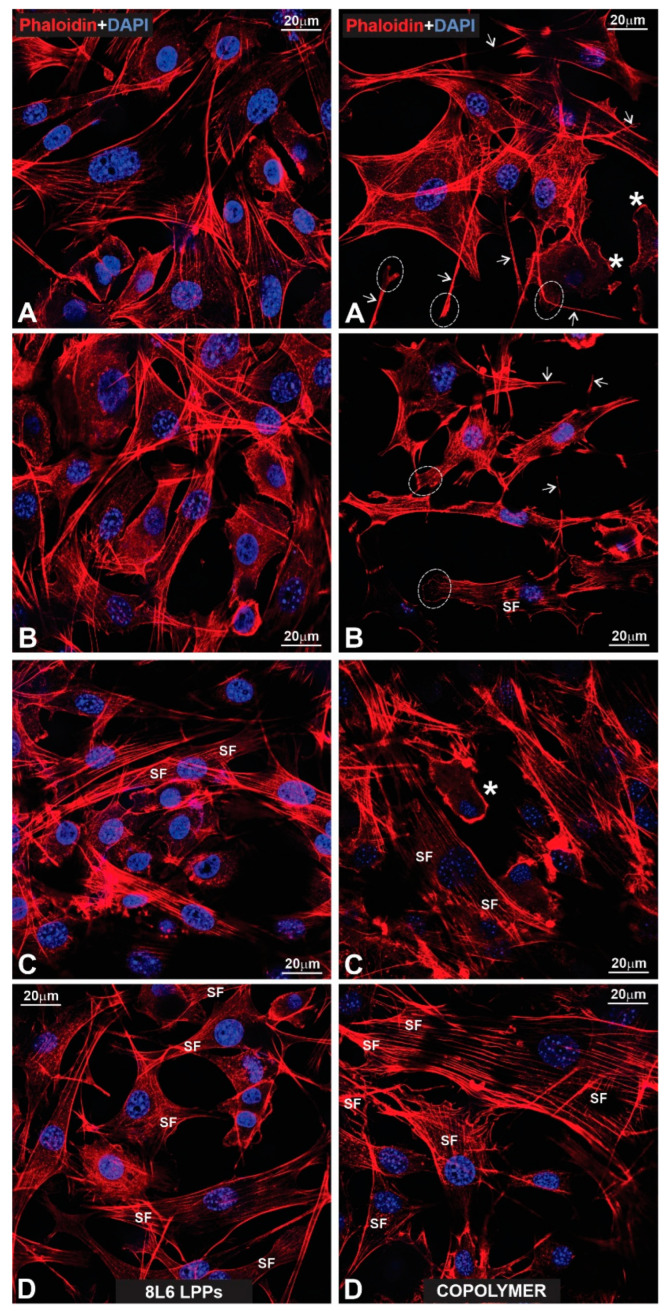
(**Left**: (**A**–**D**)) Fluorescent images of fibroblasts growing on the surface of an 8L6 PP and labeled according to the techniques shown in previous figures. Note the same stretching degree of all cells as was registered in the cases of the glass and 1L6 PP substrates. Moreover, the stress fibers (SF) reveal the same order, being organized in nearly parallel rows. (**Right**: (**A**–**D**)) Fibroblasts growing on a copolymer PP surface. All cells exhibit the same specific features as in previous images. The stars in (**A**,**C**) indicate fibroblasts that produce large lamellipodia. Long primary processes in (**A**,**B**) have been indicated with arrows, while the end-feet are outlined with dotted circles. Note that extremely stretched (“giant”) fibroblasts reveal especially profound parallel rows of stress fibers (SF).

**Figure 17:**
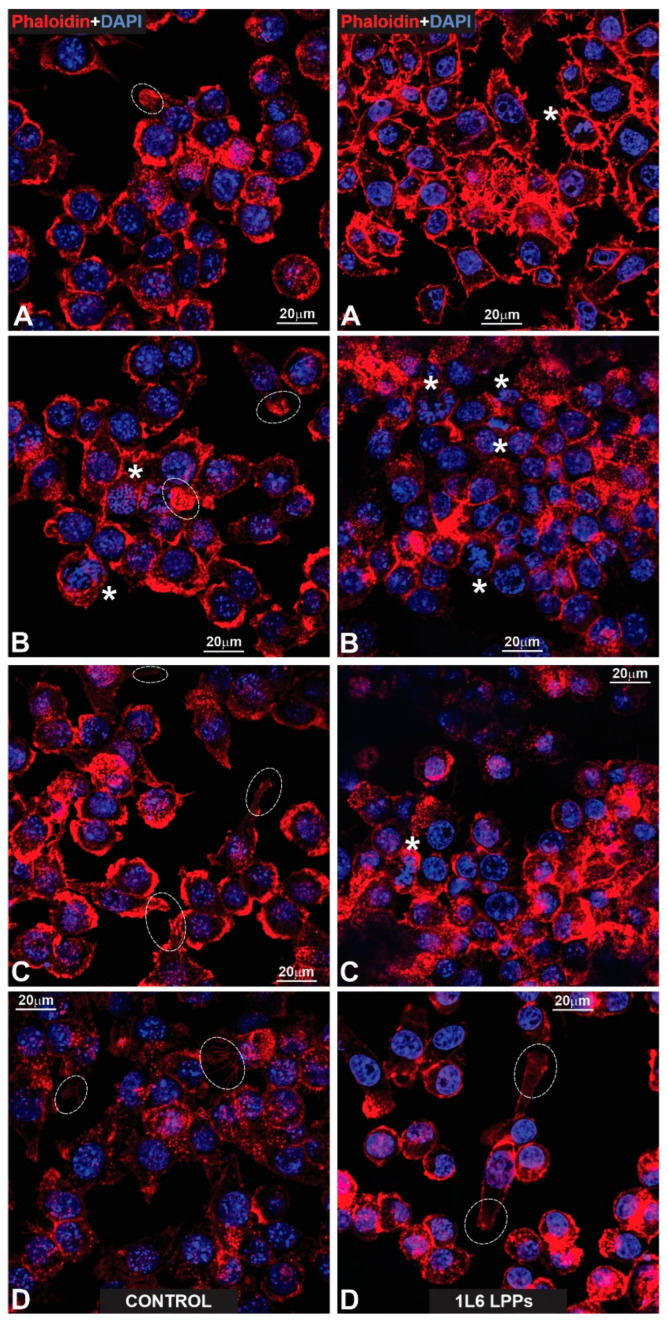
(**Left**: (**A**–**D**)) Fluorescent images presented a glass surface upon which macrophages that have been labeled for actin with phalloidin (red) and for nuclear chromatin with DAPI (blue) are growing. Note that due to the abundance of radially growing primary processes, all cells reveal a peculiar “hairy” appearance. The stars in (**C**) indicate mitotic macrophages, while their end-feet are outlined with dotted circles (**A**–**D**). Note two end-feet on the opposite poles of a spindle-like, strongly elongated macrophage (**D**). (**Right**: (**A**–**D**)) Macrophages that have been fluorescently labeled for actin (red) and nuclear chromatin (blue), according to previously applied techniques. All cells reveal a similar peculiar (“hairy”) appearance as in the control images. The stars in (**A**,**D**) indicate mitotic cells. Note the prophase macrophage that is marked with a star in (**D**).

**Figure 18:**
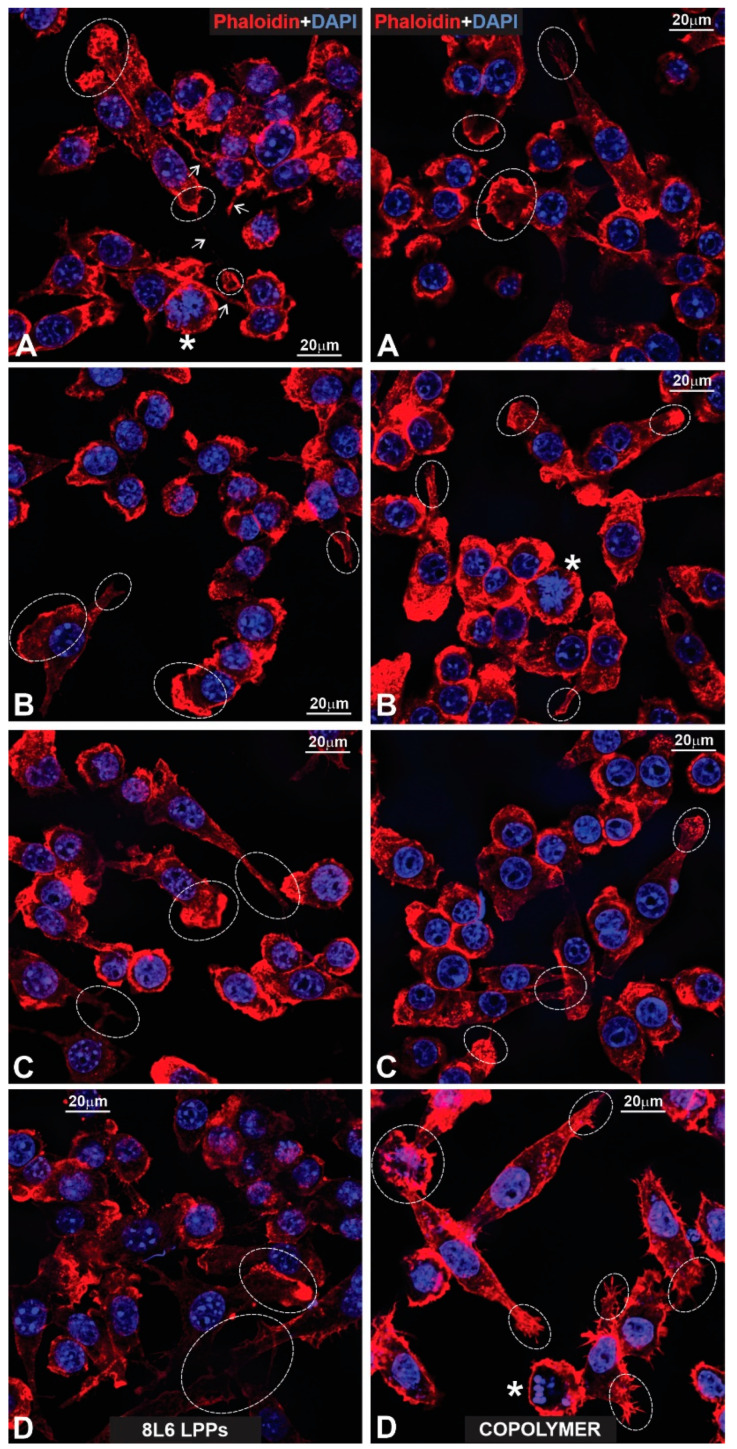
(**Left**: (**A**–**D**)) Fluorescent images of macrophages growing on the surface of an 8L6 PP. The macrophages are labeled according to the techniques shown in previous figures. Note that due to abundant primary processes, all macrophages acquired the same “hairy” appearance. The stars in images (**A**,**B**,**D**) indicate mitotic cells, while the lamellipodia in (**C**) are outlined with dotted circles. (**Right**: (**A**–**D**)) Macrophages growing on a copolymer PP surface. All cells exhibit the same peculiar “hairy” features as all previous macrophages. The arrows indicate long primary processes with growing secondary processes. The stars in (**A**,**C**) indicate mitotic cells (**A**–**D**) and a “giant” macrophage that comprises two nuclei (**A**).

To better characterize the cells’ adhesion to the LPPs, we studied the distribution of F-actin in both RAW264.7 cells and pMSFs, using Phalloidin-iFluor 647. For both cell types (Figure 15 and Figure 16—pMSFs; Figure 17 and Figure 18—RAW264.7 cells), multiple sites of focal adhesions as well as nicely distinguishable motility-related structures (like lamellipodia and filopodia) were clearly visualized, confirming the impression from the above-provided SEM images that for all three LPPs, the anchorage of the cells to the substrates appeared to be very firm.

**Figure 19:**
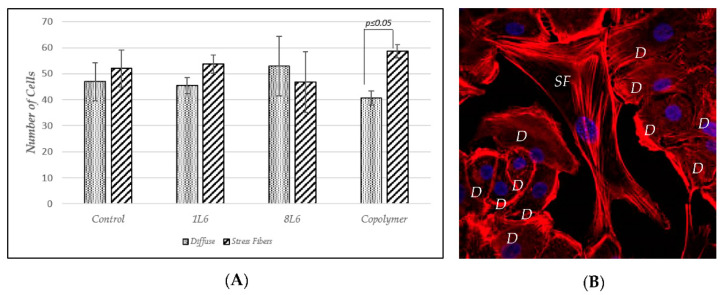
F-Actin distribution in pMSFs. (**A**) The number of cells with prevalent and diffuse distributions of F-actin vs. the number of cells with F-actin forming easily distinguishable stress fibers (the results from three independent experiments are summarized). (**B**) A representative confocal fluorescence micrograph of pMSFs stained with DAPI (blue) and Phalloidin-iFluor (red). SF—stress fibers; D—diffuse distribution of F-actin.

It is important to mention that for the fibroblasts, two different subpopulations were clearly visible: One with evenly distributed, diffuse F-actin and a subsequently diffuse distribution of chromatin within the nucleus, and a second population with numerous, clearly visible stress fibers (bundles of F-actin and myosin [3]) and nuclei containing multiple chromocenters. For each of the samples, a proportion of these distinct populations was calculated. The results are presented in Figure 19. An increase in stress-fiber/multiple chromocenter-containing cells was detected in the case of pMSFs grown on the copolymer (Figure 19). Others have shown [28,29,30,31,32] that an increase in heterochromatin that blocks the nuclei of cells is often connected to cell migration. Therefore, an increase in the number of cells containing multiple chromocenters might indicate intensified migration in the pMSFs grown on the copolymer. This corresponds well to the motility evaluation results obtained for macrophages (Figure 6). According to these results (Figure 6), the migration property of the RAW264.7 cells also increased on this LPP.

## 4. Discussion

The main goal of our experimental research was to determine whether the novel leucine-based pseudo-proteins (LPPs) 1L6 and 8L6 and a copolymer of these two LPPs (1L6_70_+ 8L6_30_) can provide appropriate support to cell cultures, and thus may be considered scaffolds for tissue engineering tasks.

For this reason, we carefully selected the following types of cells: fibroblasts (primary mouse skin fibroblasts, pMSFs) and macrophages (a murine monocyte/macrophagic cell line, RAW264.7). Fibroblasts are key extracellular matrix (ECM) producing cells; however, at the same time, they can play an opposite role by effectively degrading the ECM [26,27]. These cells can promote tissue remodeling and wound healing, and they play an important role in inflammation [26,27]. Despite being connective tissue cells, fibroblasts are present to some extent in most organismal tissues [33]. Macrophages represent another type of cell present in all tissues. Macrophages are immune cells that play a major role in both immune system stimulation and in the regulation of inflammatory processes [34,35,36]. It is important to mention that between these two types of cells, there is a reciprocal interaction that actually occurs in most tissues in states of health and disease [33]. An important common property of macrophages and fibroblasts is their morphological heterogeneity, which corresponds to their functional state [26,27,33,34,35,36]. Macrophages can be polarized toward pro-inflammatory-type cells (M1) or anti-inflammatory (pro-regulatory)-type cells (M2) [34,35,36]. M1 cells are less adhesive and less motile and have a more roundish shape, whereas M2 cells are characterized by high motility, tight adhesion, and a spindle-like (fibroblast-like) shape [34]. In the case of fibroblasts, there are also two discernable subpopulations. One subpopulation includes relatively small, spindle-like cells with diffuse distributions of F-actin and transcriptionally active chromatin [26,27]. These cells actively produce ECM degrading enzymes and participate in tissue remodeling. Another subpopulation of fibroblasts consists of highly adhesive, stretched cells characterized by the fine spreading of the cytoplasm, which contains an abundance of actin-myosin bundles—stress fibers (SF). These large, flat cells are the main ECM producing cells with inactive nuclei which contain numerous chromocenters [26,27].

Thus, for both types of cells described above, a morphological evaluation of their adhesion and growth on LPP substrates provided us with an important clue as to their functional states.

For capturing cellular morphology and its complexity in vitro, the SEM analysis has long been acknowledged as an appropriate tool for delineating cell shape, studying a cell’s surface/volume parameters, and defining cellular sociology, etc. This technique can achieve particularly accurate targets by studying cell cultures sorted for the fabrication of bioengineered cell–polymer constructs, with a special consideration given to their use in restitution and regeneration medicine. First, this field deals with the biocompatibility of new scaffolding materials, particularly in the context of cell–biopolymer structural interplay. For this reason, we performed a detailed ultrastructural analysis of the cell–polymer interfaces.

An SEM analysis of the growth of cells on the chosen LPPs revealed that all types of cultured cells adhered tightly to the surfaces of the LPPs as numerous primary and secondary processes were clearly visible (Figure 8, Figure 9, Figure 10, Figure 11, Figure 12, Figure 13 and Figure 14, Figure A1, Figure A2, Figure A3, Figure A4, Figure A5, Figure A6, Figure A7, Figure A8 and Figure A9).

It is important to stress that multiple motility-associated structures, such as lamellipodia and filopodia, have been detected in both cases of fibroblasts (pMSFs) and macrophages (RAW264.7), not only via SEM but also via confocal laser microscopy after F-actin had been stained with Phalloidin-iFluor (Figure 15, Figure 16, Figure 17 and Figure 18). An abundance of these locomotion-associated structures illustrate the high degree of motility of the cells on the LPP substrates. The migratory properties of the macrophages were separately evaluated via time-lapse microscopy and, according to the obtained results, more locomotion took place in the cases of two particular LPP films, 1L6 and the copolymer, while on the third LPP, 8L6, the cells’ motility appeared to be decreased compared to the control (Figure 6A–D). This inhibition of cell migration appeared to be due to the increased proliferation of macrophages on the surface of this particular LPP. This intensified proliferation was evident in the calculation of the mitotic index (Figure 5).

A measurement of metabolic activity in the cells grown on LPPs showed that in general, a more profound early metabolic activation of cells happened in the case of the 8L6 and copolymer substrates, while in the case of the 1L6 substrate, the metabolic activation became evident later (Figure 2). Overall, apart from this fact, all three LPPs provided high degrees of affinity toward the cell cultures and at some point, revealed a stimulatory effect; however, there was a certain dissimilarity in their specific patterns of action (e.g., time-point, intensity, etc.).

Numerous studies [3,7,9,11,37] have shown that nano/sub-micro-level surface topological properties (roughness, porosity, etc.) of a surface affect the physiology of the cells grown on the surface via the modulation of adhesive complexes that regulate cell cycle entry, cell shape, proliferation, etc. [28,29,30,31,32]. Therefore, we speculate that the dissimilarity in the LPPs’ actions in our case could be also due to the differences in the surface topologies of the studied LPPs. An SEM analysis of the LPPs’ surfaces revealed (Figure 8, Figure 9, Figure 10, Figure 11, Figure 12, Figure 13 and Figure 14, Table 1) that all three LPP films used in our experiments differ with respect to the degree of porosity and pore size, both of which were measured at a sub-micro level. This speculation needs to be confirmed at a future stage of our research.

At this stage, we can conclude that when used as substrates for cell cultures, all three LPPs exhibited clearly non-toxic and “cell-friendly” environments, allowing for excellent cell attachment and the stretching/spreading of cells on their surfaces. These findings make it clear that the suggested LPPs have good prospects for use in bio-engineering tasks.

## Figures and Tables

**Table 1:** The LPP films’ surface porosity values, measured at a sub-micro level. SEM images were analyzed using ImageJ software. The number of pores per 1^2^ μm and average pore size were calculated.

	Number of Pores per 1^2^ μm	Pore Size (in μm)
1L6	7.8 ± 0.4	0.256 ± 0.018
8L6	1 ± 0.1	0.337 ± 0.013
Copolymer	0.2 ± 0.05	0.181 ± 0.021

**Figure A2:**
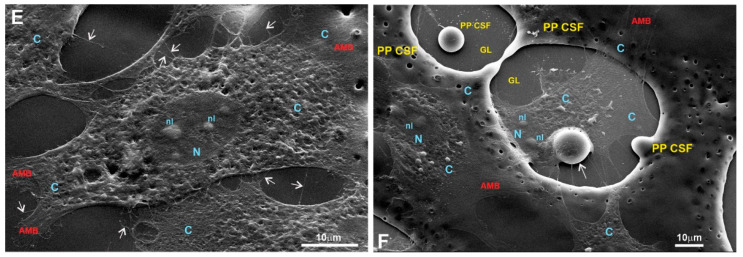
SEM images of fibroblasts growing on the surface of a 1L6 cell-supporting film (PP SCF). (**E**,**F**) High magnification. (**E**) A high-magnification image showing cell-to-cell contacts established by numerous primary and secondary processes (arrows). (**F**) The case in which the 1L6 PP deposited on cover glass forms a so-called “heterogeneous” layer, on which polymer-covered areas appear intermittent with irregular, polymer-free “holes” large enough to house cell groups on their glass (GL) bottoms. Note that the polymer-derived microsphere (arrow) could also be coated by an intensively stretched part of the cytoplasm. PP CSF—pseudo-protein cell-supporting film; C—cytoplasm; N—nucleus; nl—nucleolus; m—mitochondria; AMB—actin-myosin bundles; GL—glass.

**Figure A3:**
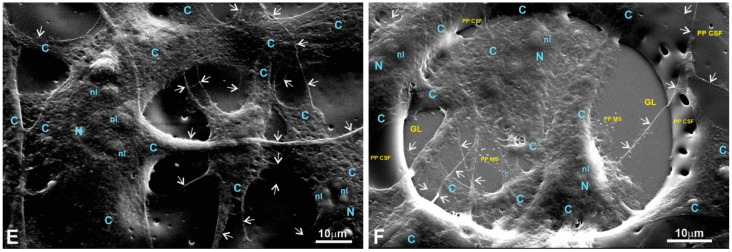
Fibroblasts growing on the surface of an 8L6 supporting film. (**E**,**F**) High magnification. (**E**,**F**) Upon closer view, it becomes evident that the cells positioned on the glass-polymer transient area (**F**) never exhibited any morphological differences, and thus the intensity of stretching on the glass and 8L6 surfaces as well as the adherence of cells to the glass and polymer film appear absolutely the same. The arrows indicate long primary processes with growing secondary processes. PP CSF—pseudo-protein cell-supporting film; C—cytoplasm; N—nucleus; nl—nucleolus; m—mitochondria.

**Figure A4:**
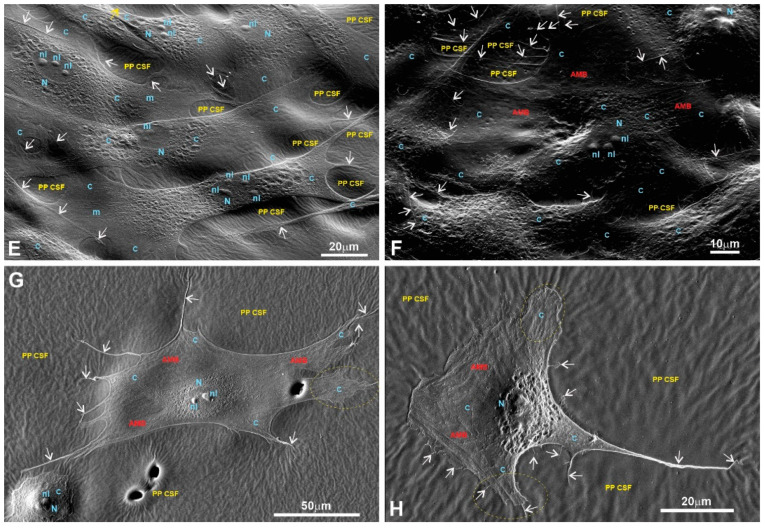
pMSFs growing on the surface of the copolymer supporting film. (**E**–**H**) Medium-magnification image focused on both cellular groups (**E**,**F**) and individual cells (**G**,**H**). Note that as a consequence of strong stretching, in all the images, the cytoplasm reveals a prominent tripartite structure (**E**,**F**). (**G**,**H**) Medium-magnification image of two separately located fibroblasts. (**G**) This strongly stretched fibroblast could simultaneously produce very long primary processes (arrows) and lamellipodia (shown in dotted circles). In the middle right part of the image, note the cytoplasmic region that focally continues inside the micro pore. (**H**) A motile fibroblast that reveals a very broad leading edge/front, with distinct end-feet (outlined with doted circles) formed on lateral sides of the large lamellipodia. The arrows indicate primary processes and multiple secondary ones. PP CSF—pseudo-protein cell-supporting film; C—cytoplasm; N—nucleus; nl—nucleolus; m—mitochondria.

## Data Availability

The data are available in a publicly accessible repository that does not issue DOIs. Publicly available datasets were analyzed in this study. These data can be found here: https://drive.google.com/drive/folders/1AjXJfwa2XjSqeS5FjyUJuE5N-HmKv7Ag?usp=sharing (accessed on 24 February 2023).

## Appendix A

Generally, two major groups of macrophages were recognizable: the first group consisted of roundish cells without remarkable cytoplasmic extensions that were not properly spread, similar to what was detected in the fibroblasts. The second group represented well-stretched, elongated cells that acquired the morphology of actively moving cells in the form of lamellipodia; regardless, they appeared settled on the glass or polymer surfaces. At a low magnification, the cells growing on the glass surface (Figure A5A–D) represent a mixed culture consisting of oval, strongly elongated, and profoundly flattened cells that were localized separately or clustered in groups of different sizes (Figure A5A,B). In addition, low-magnification images indicate that the oval-shaped macrophages formed a major population of cells. Correspondingly, by transitioning to medium and high levels of magnification, we observed mostly round or oval cells with distinctly bulged perinuclear parts of the cell. At the same time, strongly elongated/spindle-like and properly stretched cells located in close associations/neighborhoods with oval-shaped cells were also numerous. Importantly, the stretched cells exhibited typical lamellipodia at the leading edge of the motile macrophage (Figure 13E–H). Similar to fibroblasts, abundant primary and tiny secondary processes established tight contacts between the cells and between the cells and the glass. Thus, when analyzing the high-magnification SEM images, attention was paid to the major cell-specific characteristics of macrophages: the cell surfaces produced numerous depressions of different sizes that may be indicative of active endocytosis. At the same high level of magnification, the structural basis of the anchorage of the macrophages to the glass became clearly visible (Figure A5G,H).
